# Functional analysis of a novel *C*-glycosyltransferase in the orchid *Dendrobium catenatum*

**DOI:** 10.1038/s41438-020-0330-4

**Published:** 2020-07-01

**Authors:** Zhiyao Ren, Xiaoyu Ji, Zhenbin Jiao, Yingyi Luo, Guo-Qiang Zhang, Shengchang Tao, Zhouxi Lei, Jing Zhang, Yuchen Wang, Zhong-Jian Liu, Gang Wei

**Affiliations:** 1 School of Pharmaceutical Sciences, Guangzhou University of Chinese Medicine, Guangzhou, 510006 China; 2 Shantou University Medical College, Shantou, 515041 China; 3 State Key Laboratory of Systematic and Evolutionary Botany, Institute of Botany, Chinese Academy of Sciences, Beijing, 100093 China; 4 University of Chinese Academy of Sciences, Beijing, 100049 China; 5 Shenzhen Key Laboratory for Orchid Conservation and Utilization, The National Orchid Conservation Center of China and The Orchid Conservation and Research Center of Shenzhen, Shenzhen, 518114 China; 6 Department of Pharmacy, Sun Yat-Sen Memorial Hospital, Sun Yat-Sen University, Guangzhou, 510120 Guangdong China; 7 Shaoguan Institute of Danxia Dendrobium Officinale, Shaoguan, 512005 China; 8 Key Laboratory of National Forestry and Grassland Administration for Orchid Conservation and Utilization, College of Landscape Architecture, Fujian Agriculture and Forestry University, Fuzhou, 350002 China; 9 Henry Fok College of Biology and Agriculture, Shaoguan University, Shaoguan, 512005 China

**Keywords:** DNA recombination, Genetic variation

## Abstract

Flavonoids, which are a diverse class of phytonutrients, are used by organisms to respond to nearly all abiotic stresses and are beneficial for human health. Glycosyltransferase, used during the last step of flavonoid biosynthesis, is important in flavonoid enrichment. However, little is known about glycosyltransferase in the orchid *Dendrobium catenatum* (*D. officinale*). In this study, we isolated a novel *C*-glycosyltransferase (designated *DcaCGT*) from the orchid *D. catenatum* by identifying and analyzing 82 putative genes in the GT1 family. DcaCGT could specifically catalyze not only di*-C*-glycosylation but also *O-*glycosylation. Apart from the normal function of catalyzing 2-hydroxynaringenin and phloretin to the respective di-*C*-glycosides, DcaCGT also catalyzes apigenin to cosmosiin. Targeted metabolic profiling of the substrates (2-hydroxynaringenin, phloretin, and apigenin) and products (vitexin, isovitexin, vicenin-2, nothofagin, 3’,5’-di-*C*-glucosylphloretin, and cosmosiin) in different tissues showed that vicenin-2 was the most abundant product of this novel enzyme. Cosmosiin was detected in flowers and flower buds. We also established that *DcaCGT* functions expanded throughout the evolution of *D. catenatum*. Residual OGT activity may help *D. catenatum* resist drought stress. Our study illustrates the function, origin, and differentiation of *DcaCGT* and provides insights into glycosylation and molecular propagation processes, which can be used to improve the production of flavonoids by the cultivated medicinal plant *D. catenatum*.

## Introduction

Flavonoids, a class of valuable secondary metabolites, are widely distributed in plants. More than 10,000 flavonoids and their derivatives have been identified to date^1^. Flavonoid glycosides are often modified by flavonoid *O*- and *C*-glycosylation, which change the biological activities of these compounds. In plants, *C*-glycosylflavones protect against UV-B irradiation^2^, exert antimicrobial effects^3^, and are involved in plant allelopathy^4^ and copigmentation^5^. *C*-glycosylflavones are critical in plant physiology and are beneficial for human health. For example, vicenin-2 is an effective flavone that shows anti-inflammatory^6^, antiglycating^7^, antispasmodic^8^, antiseptic^9^, antiplatelet, antithrombotic^10^, and anticancer^11,12^ activity. Because flavonoids show such high biological activity, determining the pathways involved in the biosynthesis of flavonoid glycosides is important.

Glycosylation has an important role in modifying secondary metabolites, maintaining metabolic homeostasis, and detoxifying xenobiotics. These processes are mediated by a set of glycosyltransferases (GTs, EC 2.4.x.y). These enzymes are responsible for catalyzing glycosylation and are classified into 109 families according to the similarity of their amino-acid sequences in the Carbohydrate-Active enZYmes database (http://www.cazy.org). Family-1 glycosyltransferases are the largest multigene GT family, and their glycosylated products participate in the regulation of plant growth and development, such as hormone homeostasis^13^, pollination^14^, and interactions with the environment, such as detoxification of xenobiotics^15^ as well as UV tolerance^16^. GT1s are also called UDP-glycosyltransferases because they use uridine 5’-diphosphate as a sugar donor. UGTs are characterized by a highly conserved motif of a 44-amino-acid C-terminal consensus sequence called the plant secondary product glycosyltransferase (PSPG) box. Because the GT1 family has such a crucial role in the synthesis of plant secondary metabolites, members of this family have been identified mostly in model plants, crops, and fruits such as Arabidopsis (*Arabidopsis thaliana*)^17^, maize (*Zea mays*)^18^, wheat (*Triticum aestivum*)^19^, soybean (*Glycine max*)^20^, cotton (*Gossypium* genus)^21^, peach (*Prunus persica*)^22^, pear (*Pyrus bretschneideri*)^23^ and so on. However, little is known about the GT1 family in medicinal plants.

*Dendrobium catenatum* (*D. officinale*), a perennial herb in the Orchidaceae, is rich in flavonoid glycosides^24^ and is mainly distributed in Southeast, South, and Southwest China^25^. In the wild, *D. catenatum* largely grows year round on cliffs that receive abundant sunlight and scant water^26^. Owing to its high medicinal value, *D. catenatum* has been widely used in traditional Chinese medicine^27^. According to Chinese medical theory, the stems of *D. catenatum* can nourish yin, enhance gastric motility, and increase body fluids regulating immunity^28^. Modern pharmacological studies have shown that the active ingredients of *D. catenatum* include polysaccharides^29^, flavonoids^26^, and bibenzyls^30^, which promote salivation and relieve the symptoms of diabetes^31,32^.

Glycosylation is the final step in the synthesis of flavonoid glycosides. Using oxygen, *O*-glycosyltransferases link the *O*-glycosylflavone sugar moiety to the flavonoid skeleton, whereas the glucose moiety of *C*-glycosylflavones is directly bound to the flavone backbone. *C*-glycosylflavone biosynthesis is catalyzed by *C*-glycosyltransferases (CGTs). In 2009, CGTs were first reported in rice (OsCGT)^33^, followed by maize (UGT708A6)^34^, buckwheat (FeCGTa, FeCGTb)^35^, and soybean (UGT708D1)^36^. Most *C*-glycosylflavones are synthesized via glucosylation of the substrate 2-hydroxyflavanone, followed by dehydrase-mediated dehydration. In 2014, another GtUF6CGT-mediated *C*-glycosylation pathway was identified in *Gentiana triflora*, showing that the sugar moiety can be directly catalyzed to the flavone skeleton^37^. In 2017, the first CGT catalyzing the glycosylation of di-*C*-glycosides was found in citrus fruits (UGT708G1, UGT708G2)^38^. In 2019, the first flavone 8-*C*-glycosyltransferase was discovered in *Trollius chinensis* (TcCGT)^39^.

Di-*C*-glycosylflavones are the main flavonoids in *D. catenatum*^40^. However, the amounts of secondary metabolites in *D. catenatum* vary regionally. Higher levels of di-*C*-glycosylflavones may help this plant adapt to a harsh growing environment and affect the levels of medically important ingredients. Although the last step in the formation of *C*-glycosylflavones is well characterized in plants, the gene involved in the biosynthesis of di-*C*-glycosylflavones in *D. catenatum* has not been identified. In this study, we performed a genome-wide analysis and functional verification of CGT genes in *D. catenatum*. The levels of *DcaCGT*-related flavonoids were evaluated by ultra-high-performance liquid chromatography coupled with triple-quadrupole mass spectrometry. The haplotype distribution of *DcaCGT* was assessed in 274 samples acquired from 34 populations. Our study provides the biosynthetic pathways of di-*C/O*-glucosylflavones in *D. catenatum*, and performs a molecular phylogenetic analysis to offer insight into the evolution of the functional gene.

## Results

### Identification of *UGT* genes in *D. catenatum*

**Fig. 1:**
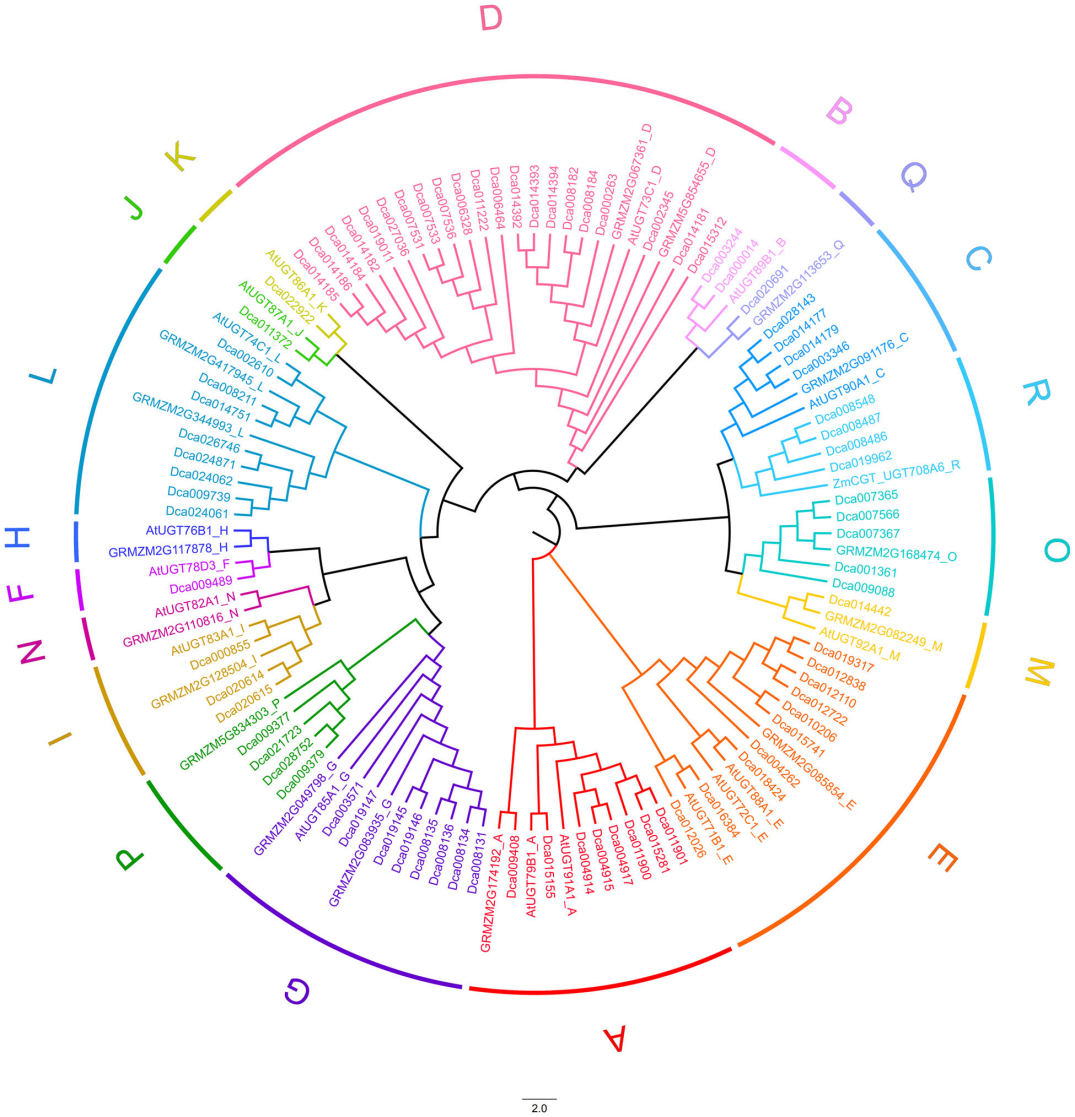
Phylogenetic analysis of *D. catenatum UGT* family genes. The neighbor-joining tree was created by MEGA5 software (bootstrap-value: 10,000) with full-length amino-acid sequences of 82 Dca UGTs and 34 functionally characterized UGTs, including 17 Arabidopsis UGTs and 17 maize UGTs. (Accession numbers are presented in Table S2)

A BLASTP search of the *D. catenatum* genome (GenBank accession number: PRJNA262478)^29^ was performed using the UGT-conserved PSPG box sequence as a query. A total of 82 *D. catenatum* UGTs (*E* value < 10^−5^) having lengths of 297–609 amino acids (with an average length of 471 amino acids) were identified. A phylogenetic tree of *D. catenatum* UGT genes was constructed by aligning the full-length amino-acid sequences of *D. catenatum* UGTs with functionally characterized plant UGTs, including Arabidopsis and maize UGTs (Fig. 1). These *D. catenatum* UGTs were phylogenetically divided into 16 groups, including 12 groups (A-G, I–M) that were identified in Arabidopsis^17^, 3 groups (O-Q) that were identified in maize^18^ and 1 group (R) that was found in seed plants^41^. Among the 82 listed UGTs, few were functionally characterized. The predicted molecular weight ranged from 33 to 68 kDa. The isoelectric point ranged from 4.79 to 8.9 (Table S1). Based on homology analysis, CGT gene candidates were selected from the 82 putative *UGTs*.

### Chromosomal distribution of *D. catenatum UGT* genes

**Fig. 2:**
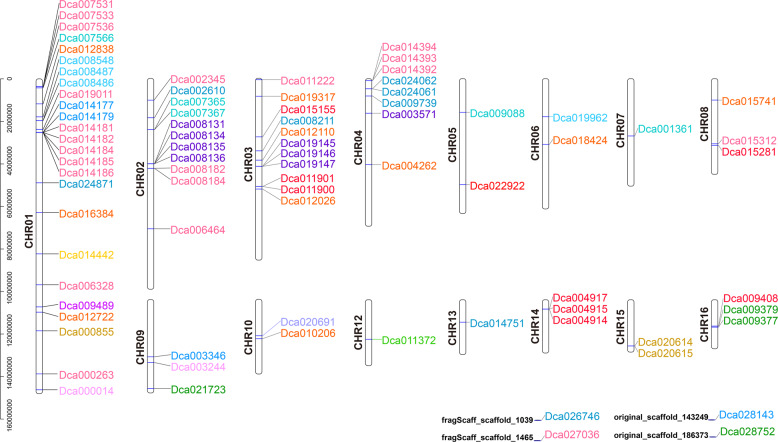
Chromosome distribution of *D. catenatum UGT* genes. Chromosome numbers are shown to the left of each chromosome. Different colors indicate the different phylogenetic groups of *D. catenatum UGT* genes

The genomic distribution of each *UGT* is shown in Fig. 2 to provide an overview of the location of *D. catenatum UGT* genes. The *D. catenatum* genome contained 19 chromosomes, but the 82 *UGTs* were distributed across only 15 chromosomes (Fig. 2). The four *UGTs* on the low right side of the figure are located on scaffolds and include *Dca026746*, *Dca027036*, *Dca028143*, and *Dca028752*. Different members of the *UGT* family were observed on each chromosome. Twenty-five *UGTs* were located on the longest chromosome, 01, followed by 11 members each on chromosomes 02 and 03 and 8 *UGTs* on chromosome 04. In addition, 1–3 *UGTs* were distributed on the other 11 chromosomes. Members of the same group were observed on several different chromosomes. *UGTs* belonging to the same group on the same chromosome tended to cluster together, whereas others were clustered separately. Groups D and E, containing the highest and second highest number of genes, respectively, were randomly distributed across five different chromosomes (Table S1).

### Sequence analysis of *D. catenatum UGT* genes

**Fig. 3:**
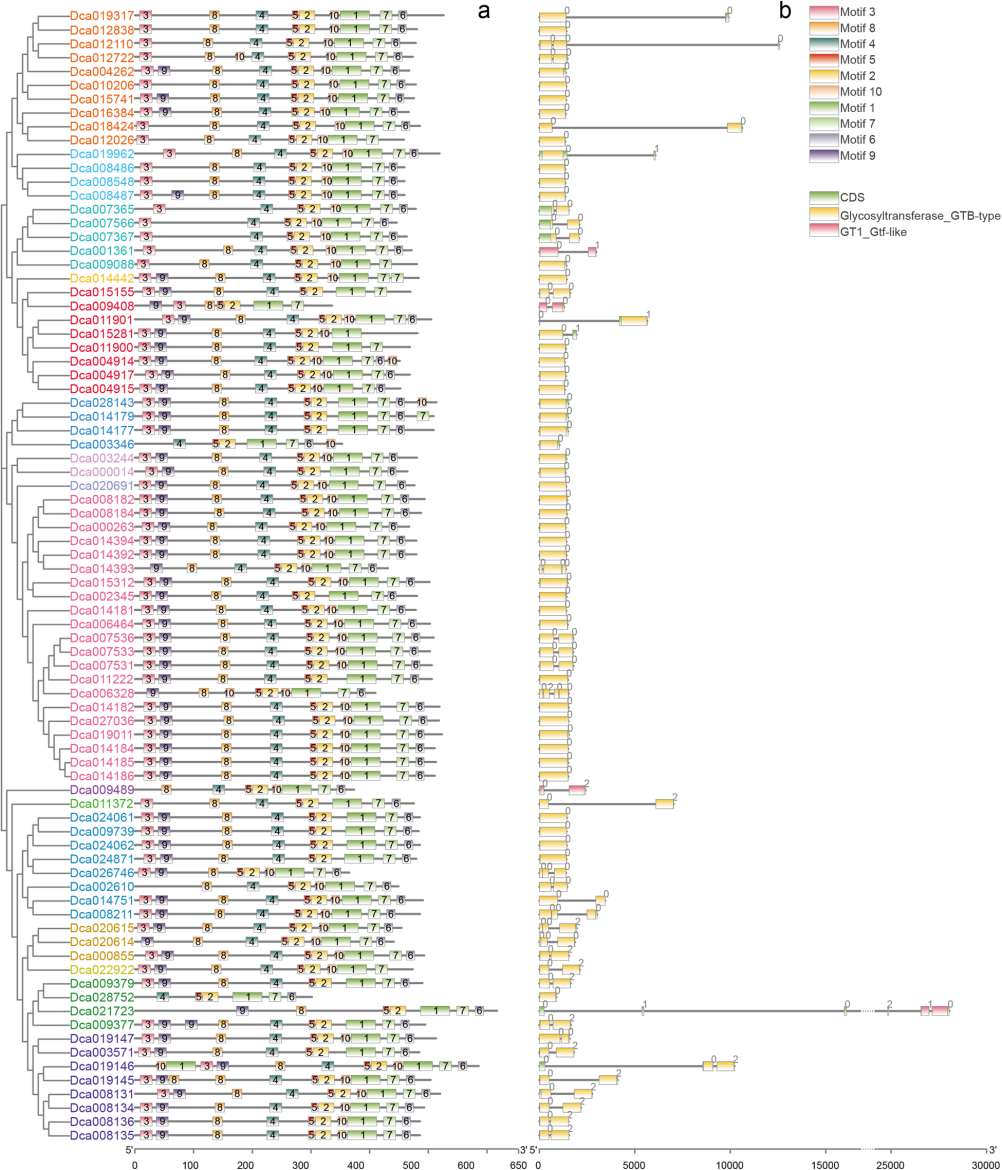
Gene structure and architecture of conserved protein motifs in *UGT* family genes of *D. catenatum*. **a** Motif composition of UGTs; colored boxes indicate 10 different motifs. **b** Exon–intron structure and NCBI CD search results of *UGT* genes. Colored boxes indicate exons; black lines indicate introns; and numbers indicate the corresponding intron phases

Next, we analyzed exon–intron organization to investigate the evolutionary relationships within the *D. catenatum UGT* gene family. Among the 82 *UGTs*, 43 contained no introns, and 30 contained one intron in each gene. For the remaining nine *UGTs*, seven contained two introns, and only one *UGT* each contained three and five introns. *UGTs* without introns occurred more commonly than those with introns. For the *UGT* groups, 100% intron loss was observed in genes of groups B, C, and Q, followed by 76% of genes in group D and 60% of genes in group M. Conversely, all genes in groups F, G, H, I, and J contained introns. A total of seven (87%) *UGTs* in group G contained one intron. These positions of intron insertion were determined to be highly conserved after mapping the introns via alignment of amino-acid sequences. Intron-insertion positions varied in groups A, E, O, and R. Among the 52 total introns detected in *D. catenatum UGT* sequences, 48, 2, and 2 were in phases 0, 1, and 2, respectively. In addition, 92% of introns were in phase 0, which is far greater than the number in phases 1 and 2. Based on the information presented above, introns in *D. catenatum* family-1 UDP-glycosyltransferase clusters were deemed to not be completely conserved. In addition, an NCBI CD search showed that most of the 82 *UGTs* were GTB-type glycosyltransferases (Fig. 3b).

A schematic representation of the structure of all *D. catenatum* UGT proteins was constructed from the results of MEME motif analysis. As shown in Fig. 3a, motifs 1, 2, and 5 were widely distributed in every gene. Motif 1 was the location of the PSPG box, whereas the protein architecture of motifs 2 and 5 was speculated to be conserved in *D. catenatum* UGTs. Members within the same groups were usually found to share similar structures. For example, all members in group O, three of four genes in group R and 7 of 10 genes in group E showed the absence of motif 9. Three-fifths of the UGTs in group O showed the loss of motif 8. Together with the results of the phylogenetic analysis, this similarity in motif arrangements among *D. catenatum* UGT proteins within subgroups indicates that the subfamily classification of UGTs may be based on partially conserved protein domains.

### Gene expression analysis of different organs of *D. catenatum*

**Fig. 4:**
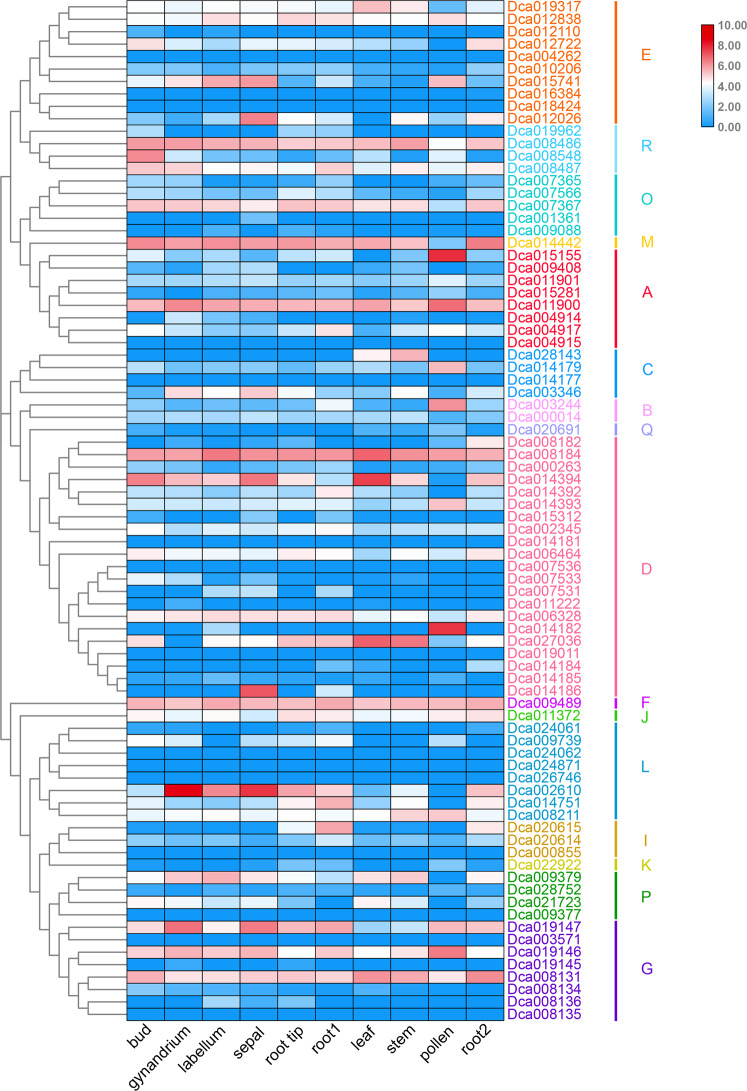
Expression profiles of *UGT* genes in different organs. Expression potential from low to high is denoted by square colors from blue to red. Different letters on the right side of the figure indicate different phylogenetic groups of *D. catenatum UGT* genes

The transcriptome revealed 82 *UGTs* with varied expression patterns across 10 plant tissues (Fig. 4), including the bud, gynandrium, labellum, sepal, root tip, day root (root1), leaf, stem, pollen, and night root (root2) (GenBank accession number: PRJNA348403)^42^. Sixty-nine *UGT* genes were expressed in at least two organs, whereas transcription of 13 *UGTs* was not observed. For the unexpressed *UGTs*, three genes were located in groups D and L, two genes were located in group E, and the other six genes were distributed among groups A, C, G, I, and P. We examined the possible expression pattern of *UGTs* in different tissues and found that 58 *UGTs* were expressed in the bud and sepal, which were the organs showing the highest number of expressed *UGTs*. The lowest number of *UGTs* (49*)* was found in pollen tissue. The number of *UGTs* expressed in other organs, such as the gynandrium, labellum, root tip, day root, leaf, stem, and night root, ranged between 49 and 58. We also observed that unexpressed genes in these tissues were mostly distributed in groups D, G, L, and E. In contrast to 35 *UGTs*, which were expressed in all nine plant tissue samples, nine *UGTs* were expressed in all but one organ sample. *Dca027036* and *Dca015281* expression was not detected in the gynandrium, that of *Dca015155* and *Dca012026* was not detected in leaves, that of *Dca009379*, *Dca014392*, *Dca012722*, and *Dca002610* was not detected in pollen, and that of *Dca008134* was not detected in the night root. These results suggest that high transcription levels of a specific *UGT* may lead to increased levels of related components, whereas unexpressed *UGTs* in different organs may result in the absence of certain components in these organs.

### Properties of recombinant *D. catenatum* CGT expressed in *E. coli* and in vitro enzymatic activity assays

To determine the functions of *DcaCGT*, full-length cDNA was obtained from *D. catenatum*. The CDS region was cloned into a pET32a(+) vector and transformed into *Escherichia coli* Rosetta (DE3) to investigate the catalytic activity of the enzyme encoded by the isolated putative *DcaCGT* gene (GenBank accession number: MT452646). The expressed recombinant proteins contained a fused His6 tag and showed a molecular weight of 66.1 kDa when analyzed via SDS-PAGE (Fig. S1). Enzymatic activity assays were conducted using three flavanone substrates: 2-hydroxynaringenin, phloretin, and apigenin. Glycoside products were analyzed by liquid chromatography–mass spectrometry (LC-MS).

**Fig. 5:**
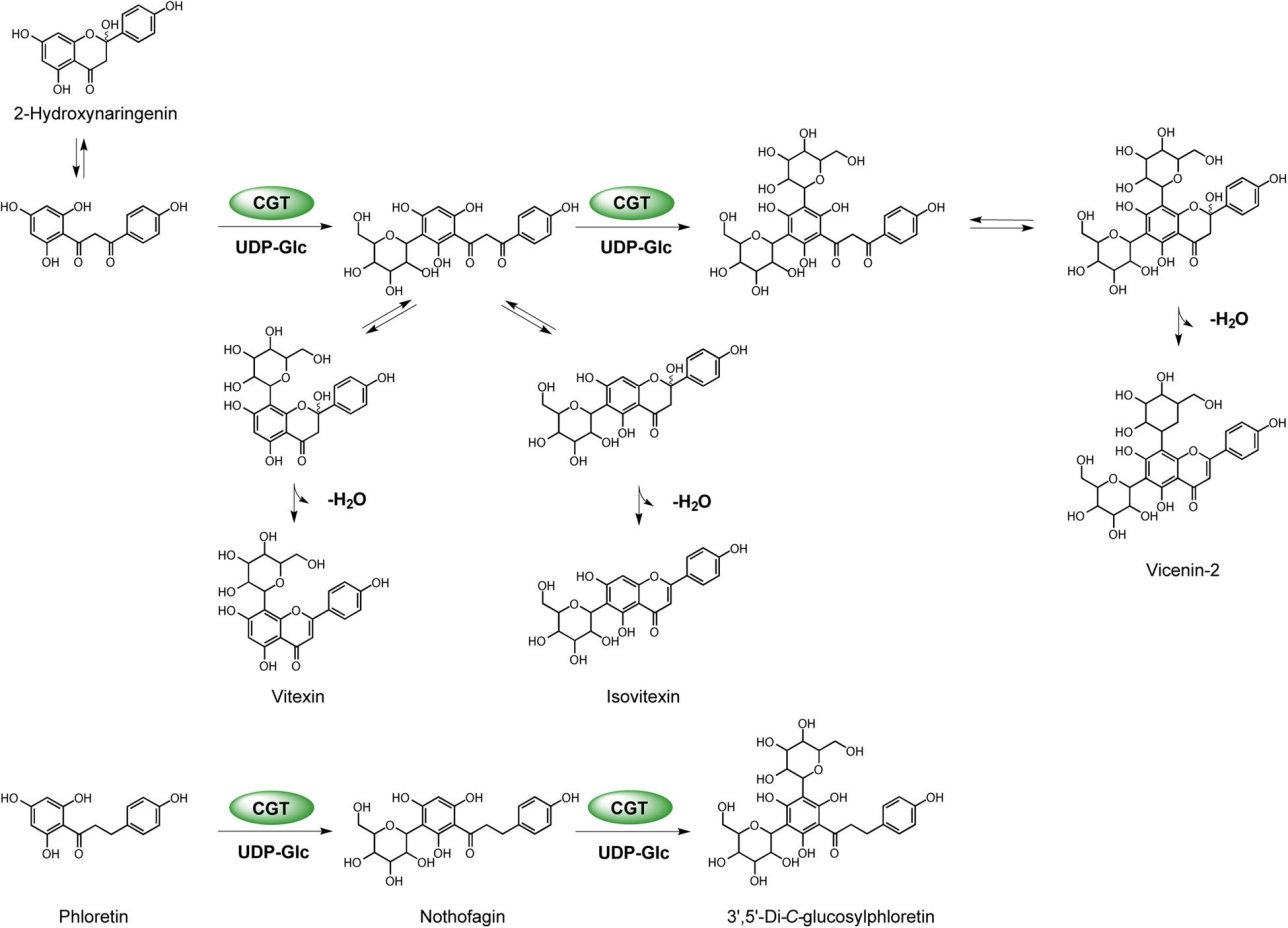
Proposed biosynthetic pathways of di-*C*-glucosylflavone in *D. catenatum*. **a** Vicenin-2; **b** 3’,5’-di-*C*-glucosylphloretin

**Fig. 6:**
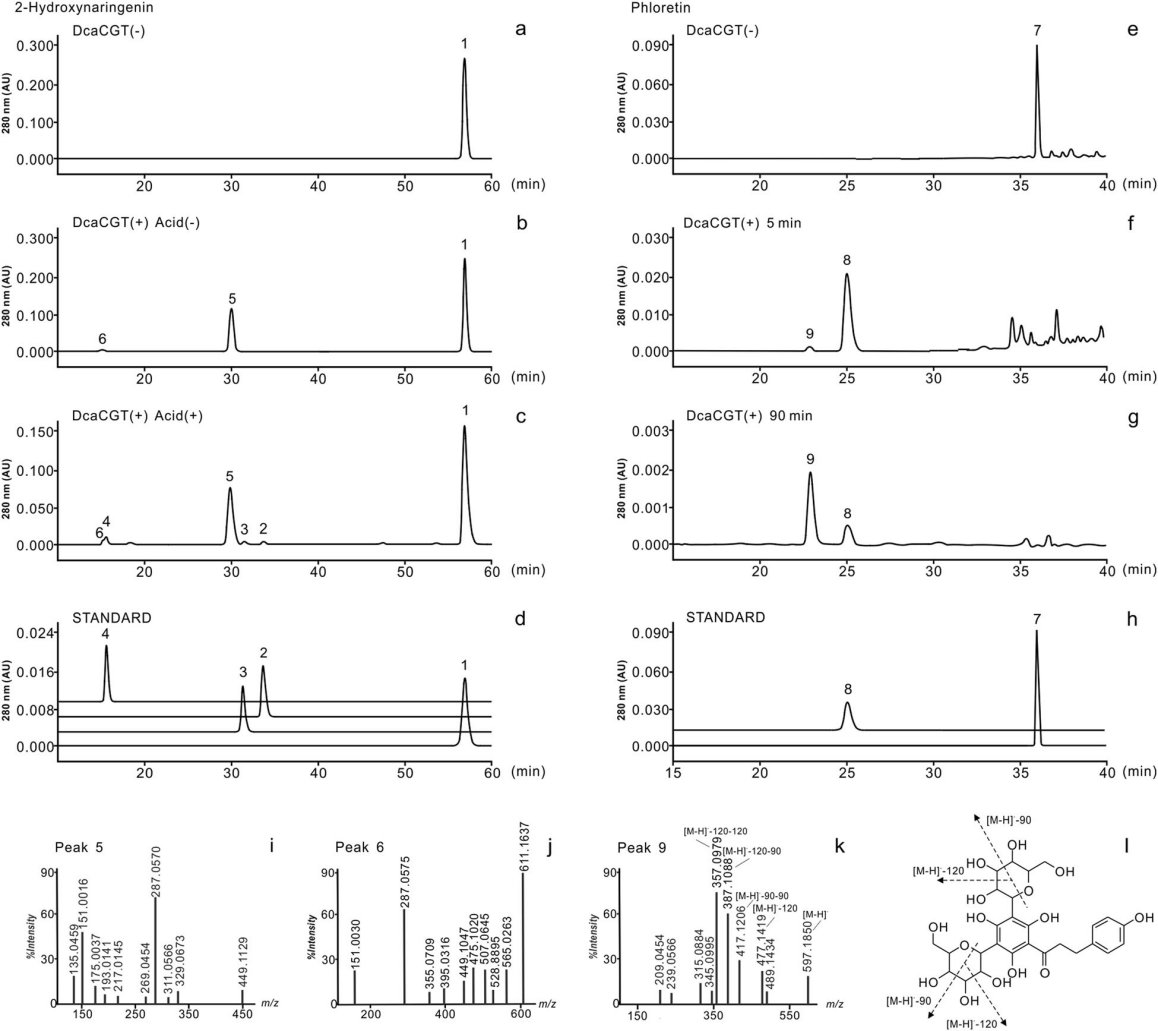
HPLC-MS analysis of DcaCGT *C*-glucosyltransferase reactions using 2-hydroxynaringenin and phloretin as substrates. Cells on the left show chromatograms of the enzymatic activity assays using 2-hydroxynaringenin as a substrate **a** with inactivated protein; **b** with activated protein before acid treatment; and **c** with activated protein after acid treatment. **d** Standards for related compounds: 1, 2-hydroxynaringenin; 2, isovitexin; 3, vitexin; 4, vicenin-2. Cells on the right show chromatograms for the enzymatic activity assays using phloretin as a substrate **e** with inactivated protein; **f** incubated for 5 min; and **g** incubated for 90 min. **h** Standards for related compounds: 7, phloretin; 8, nothofagin. MS^2^ spectra for the proposed intermediate products: **i** 5, 6-C-glucosyl-2-hydroxynaringenin; **j** 6, 6,8-di-C-glucosyl-2-hydroxynaringenin. **k** MS^2^ spectra and **l** fragmentation of the proposed product 3’,5’-di-*C*-glucosylphloretin

The resulting recombinant protein DcaCGT displayed *C*-glucosylation activity against 2-hydroxynaringenin, indicating that it was a CGT derived from *D. catenatum* plants. The typical reaction pattern catalyzed by DcaCGT is shown in Fig. 5. When 2-hydroxynaringenin was used as the substrate, it was converted into 6-*C*-glucosyl-2-hydroxynaringenin at m/z 449 (peak 5) and 6,8-di-*C*-glucosyl-2-hydroxynaringenin at m/z 611 (peak 6); these were the main products following the enzymatic reaction (Fig. 6). After treatment with HCl, these compounds were dehydrated. Two products of dehydrated derivatives (peaks 2 and 3) showed *C*-glycoside moiety fragment ions of [M-H-90]^−^ and [M-H-120]^−^^43^, which corresponded to vitexin (m/z 431) and isovitexin (m/z 431), respectively. Vitexin and isovitexin levels decreased as the reaction continued, whereas the level of vicenin-2 (m/z 593) increased (Fig. S2). All the dehydrated products showed the same retention time and fragmentation pattern as those of the respective standards^44^. Moreover, two products were observed when phloretin was used as the substrate. The MS^2^ spectrum of peak 8 at m/z 345 [M-H-90]^−^ and 315 [M-H-120]^−^ showed the presence of nothofagin^45^. Peak 9 showed a pseudomolecular ion at m/z 597. The fragments at m/z 477 [M-H-120]^−^, 417 [M-H-90–90]^−^, and 357 [M-H-120–120]^−^ suggested that most of these were gained from the loss of sugar residues (Fig. S3). Based on ESI-MS data, this product was identified as 3ʹ,5ʹ-di-*C*-glucosylphloretin^46^. These results indicate that *DcaCGT* encodes a glycosyltransferase that catalyzes the conversion of flavanones to flavonoid di-*C*-glucoside via flavonoid mono-*C*-glucoside intermediates.

**Fig. 7:**
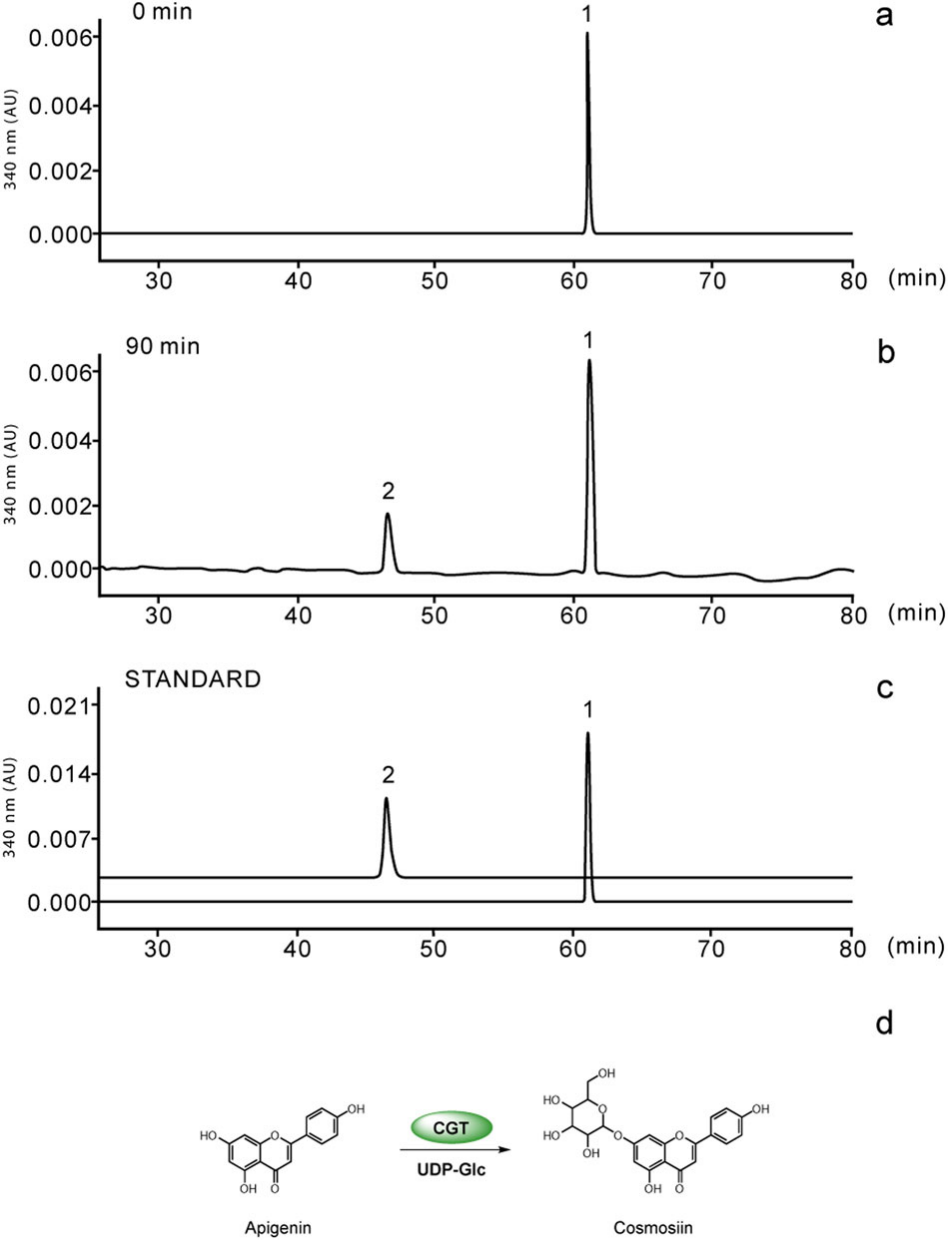
HPLC-MS analysis and proposed pathway of DcaCGT *O*-glucosyltransferase reactions with apigenin. The cells show chromatograms for the enzymatic activity assay using apigenin as substrate and incubations for **a** 0 min and **b** 90 min. **c** Standards for related compounds: 1, apigenin; 2, cosmosiin. **d** Proposed biosynthetic pathway for *O*-glucosylflavone in *D. catenatum*

In addition, when we used apigenin as a substrate, a new product was detected by LC-MS (Fig. 7). MS analysis showed a precursor at m/z 431 (Fig. S4). The fragments at m/z [M-H-162]^−^ indicated apigenin bonding to glucose via *O*-linkage^47^. The new product exhibited the same retention time and mass fragmentation pattern as those of the authentic standard for cosmosiin. Hence, these results support those obtained using bioconversion assays and indicate that DcaCGT can catalyze substrates to not only di-*C*-glycosylflavone but also *O*-glycosylflavone.

### Flavonoids of *D. catenatum*

**Fig. 8:**
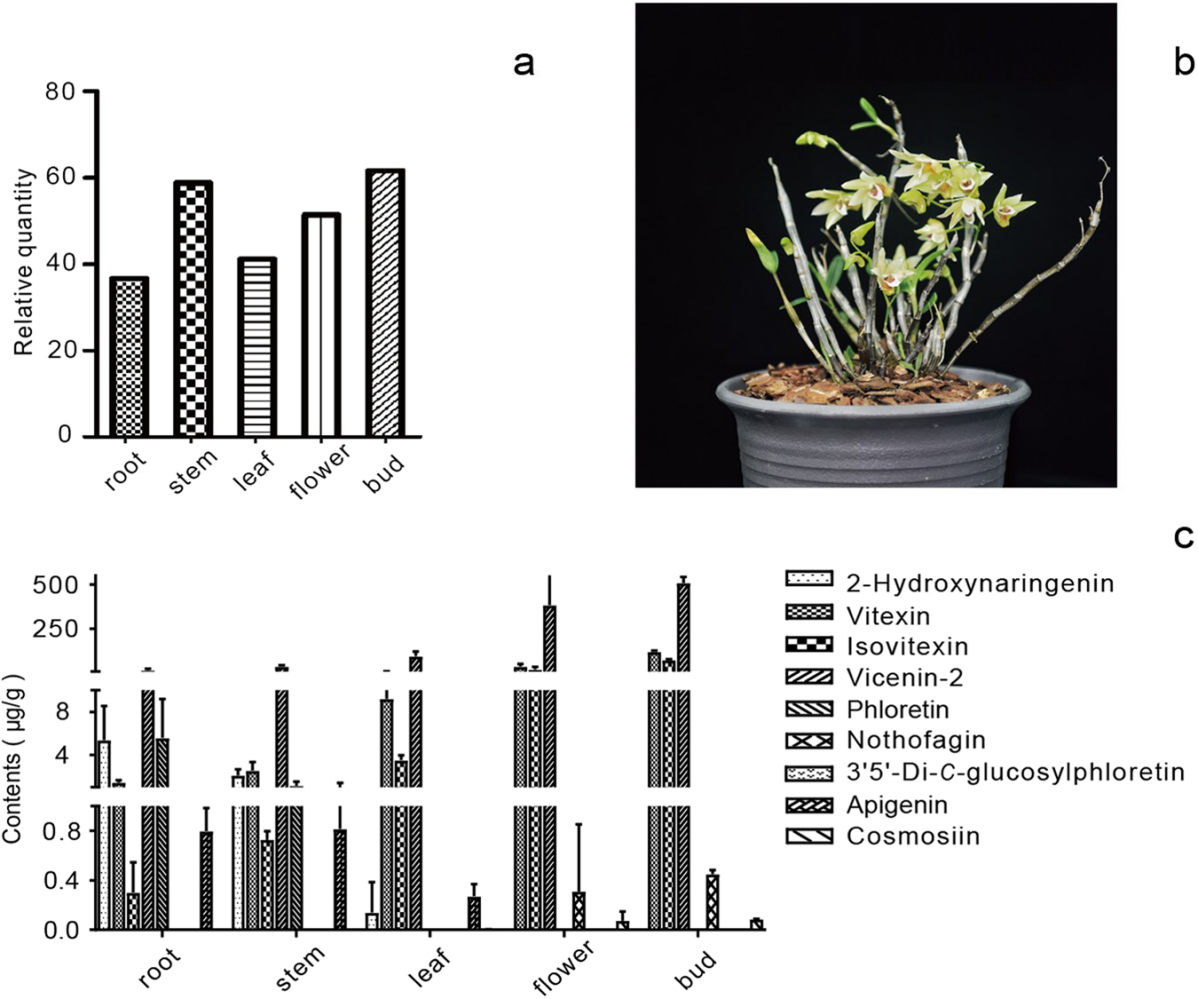
Expression pattern of DcaCGT and flavonoid contents in different organs of *D. catenatum*. **a***DcaCGT* expression pattern examined by transcriptomic analysis; **b** representative images of *D. catenatum* tissue used for metabolic profiling; **c** flavonoid contents assessed by LC-MS

The biosynthesis of flavones (vitexin, isovitexin, vicenin-2, nothofagin, 3’,5’-di-*C-*glucosylphloretin, and cosmosiin) from flavanones (2-hydroxynaringenin, phloretin, and apigenin) is catalyzed by UDP-glycosyltransferases. To explore the distribution of UGT-related flavanones and flavone glycosides accumulated in *D. catenatum*, we prepared methanolic extracts from flower buds, flowers, leaves, stems, and roots for UPLC-MS analysis. The chromatogram and mass spectrometry conditions of the eight investigated compounds as well as the internal standard are shown in Fig. S5 and Table S4. Among the nine targeted flavonoids, eight compounds (except 3’,5’-di-*C-*glucosylphloretin) were found in the different organs. The compositions of these compounds varied among the organs (Fig. S6). *C*-glycosylflavone accumulation was significantly higher than that of *O*-glycosylflavones in the plant tissues examined in this study. Vicenin-2 and its intermediate products were more abundant than nothofagin and its intermediate products. 2-Hydroxynaringenin, phloretin, and apigenin were mostly detected in the roots, stems, and leaves, while vicenin-2, vitexin, isovitexin, nothofagin, and cosmosiin showed higher levels in flowers and flower buds. Flower buds showed the greatest variety of flavone glycosides and included vitexin, isovitexin, and vicenin-2, whereas roots had the lowest levels of vicenin-2 and intermediate products. These results, together with those of transcriptomic analysis, indicate that DcaUGT may mainly be used to biosynthesize vicenin-2 and that higher levels of vicenin-2 may be related to higher *DcaUGT* transcript levels in *D. catenatum*. Other substrates and products, such as apigenin and cosmosiin, were not detected simultaneously in the same organ. This may be related to different periods of gene expression and harvest times (Fig. 8 and Table 1).

**Table 1:** Flavonoids in different organs of *D. catenatum*

Name	RT (min)	Formula	Parent/product ion	Flavonoid contents ($\overline x \pm s$, μg/g, *n* = 3)				
				Root	Srem	Leaf	Flower	Flower bud
2-Hydroxynaringenin	8.59	C_15_H_12_O_6_	286.97/151.00	5.39 ± 3.17	2.11 ± 0.56	0.14 ± 0.24	n.d.	n.d.
Vitexin	5.81	C_21_H_20_O_10_	430.95/311.00	1.46 ± 0.20	2.55 ± 0.80	9.21 ± 2.17	38.88 ± 13.69	121.19 ± 6.95
Isovitexin	6.03	C_21_H_20_O_10_	430.88/311.00	0.30 ± 0.24	0.73 ± 0.07	3.53 ± 0.45	21.36 ± 15.36	74.65 ± 4.93
Vicenin-2	3.64	C_27_H_30_O_15_	592.99/352.90	16.90 ± 8.34	38.61 ± 6.97	95.74 ± 28.07	383.52 ± 187.65	507.95 ± 35.24
Phloretin	9.12	C_15_H_14_O_5_	273.740/167.80	5.59 ± 3.60	1.15 ± 0.39	n.d.	n.d.	n.d.
Nothofagin	6.80	C_21_H_24_O_10_	435.09/314.90	n.d.	n.d.	n.d.	0.31 ± 0.62	0.45 ± 0.03
Apigenin	9.26	C_15_H_10_O_5_	268.76/117.10	0.80 ± 0.19	0.82 ± 0.61	0.27 ± 0.10	n.d.	n.d.
Cosmosiin	7.58	C_21_H_20_O_10_	430.76/268.00	n.d.	n.d.	n.d.	0.08 ± 0.08	0.09 ± 0.00

*n.d.* not detected.

### Molecular phylogenetic analysis of *D. catenatum* CGTs

**Fig. 9:**
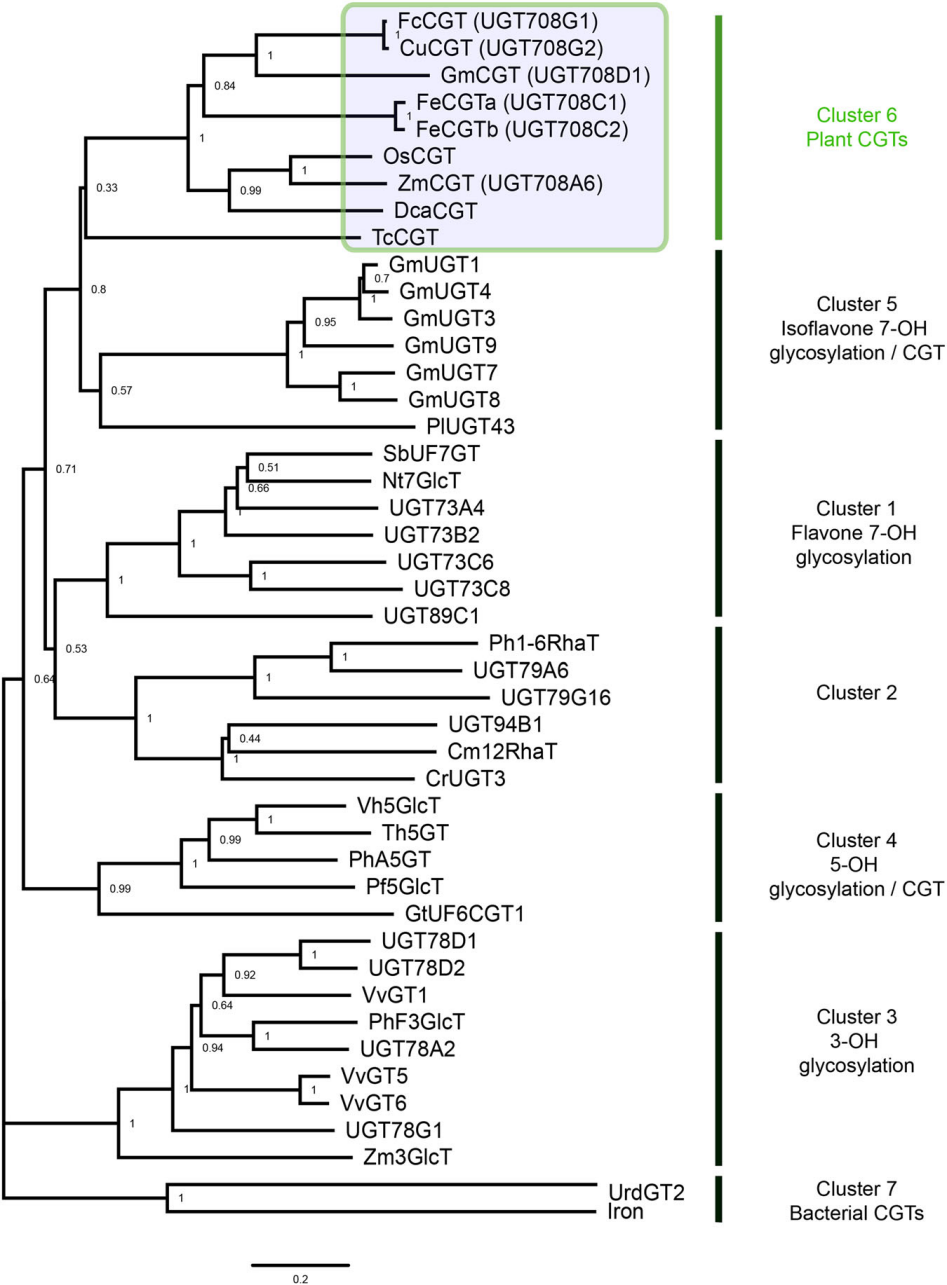
Neighbor-joining tree of DcaCGT and related glycosyltransferases. Phylogenetic analysis was performed by MEGA 7.0 using the neighbor-joining method and carried out via the bootstrapping method with 1000 replicates. The UGT amino-acid sequences were aligned using ClustalW. (Accession numbers are presented in Table S5)

A neighbor-joining tree was constructed based on the deduced amino-acid sequences of the seven clusters (Fig. 9) and included flavone 7-OH glycosylation (cluster 1), flavonoid glycoside sugar-*O*-sugar links (cluster 2), 3-OH glycosylation (cluster 3), 5-OH glycosylation (cluster 4), isoflavone 7-OH glycosylation (cluster 5), plant CGTs (cluster 6), and bacterial CGTs (cluster 7). Detailed information on these UGTs is summarized in Table S5. DcaCGT is listed in cluster 6 with other plant CGTs, indicating that they may have evolved from the same ancestral gene. DcaCGT and ZmCGT were grouped into a clade, suggesting that they may have similar active sites used to catalyze flavonoid *C*-glycosides and *O*-glycosides simultaneously. Alignments of the amino-acid sequences of DcaCGT and other plant *C*-glycosyltransferases are shown in Fig. S7.

### Multiple sequence alignment analysis of *DcaCGT* from *D. catenatum* collected from different geographical locales

**Fig. 10:**
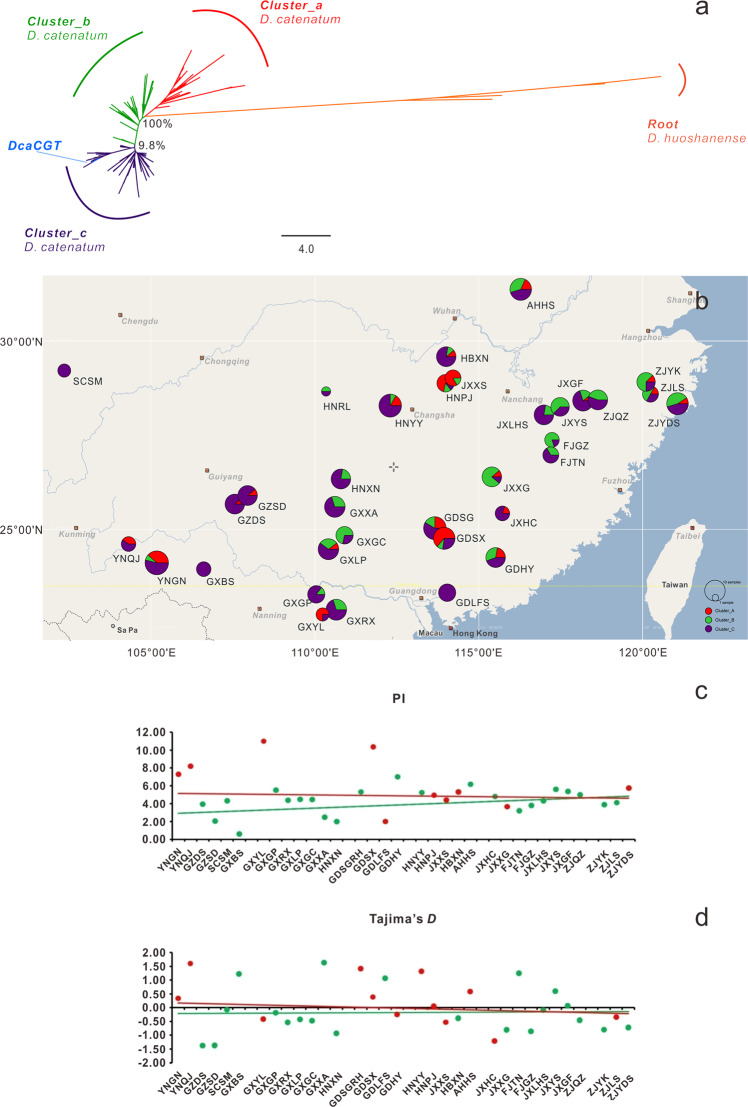
Phylogenetic, geographic, and diversity analyses of *DcaCGT* in populations of *D. catenatum*. **a** Neighbor-joining tree. Bootstrap-values for the clusters are 100 and 9.8%. **b** Geographic distribution of 274 *D. catenatum* accessions with three main clusters of 132 haplotypes. **c** Pi and **d** Tajima’s *D* values of 33 populations. Red dots: ancestral populations; green dots: derived populations; red line: trendline with ancestral populations; green line: trendline without ancestral populations (ancestral populations: Cluster_a/Cluster_abc ≥15%)

To examine the evolutionary origin of *DcaCGT*, full-length *DcaCGT* was extracted and analyzed from 279 accessions of *Dendrobium*, including resequencing data from 274 *D. catenatum* samples obtained from 34 populations and data from five samples of *D. huoshanense* obtained from a single population. As many as 132 haplotypes were observed among the aligned sequence data from the 274 *D. catenatum* samples. The *D. huoshanense* population was chosen as the root. Phylogenetic analysis indicated that *DcaCGT* haplotypes were grouped mainly into three clusters: the ancestral Cluster_a and evolutionary clade Clusters_b and _c, with 100 and 9.8% bootstrap support, respectively (Fig. 10a). In addition, the 274 samples were unevenly distributed among the three clades, with Cluster_a containing 46 samples (32 haplotypes), Cluster_b containing 72 samples (36 haplotypes), and Cluster_c containing 156 samples (64 haplotypes). A comparison of nucleotide diversity among these three clusters indicated that Cluster_a (*θ* = 0.00752; *π* = 0.00461) exhibited higher diversity than Cluster_b (*θ* = 0.00576; *π* = 0.00244) and Cluster_c (*θ* = 0.00705; *π* = 0.00287). Negative Tajima’s *D* values were observed among these clades. However, significant negative Tajima’s *D* values were only observed in the evolutionary clades for Cluster_b and Cluster_c. *DcaCGT*, the CGT identified in this study, was grouped in Cluster_c. These results suggest that natural selection acted on the evolutionary clades. Significant differentiation was found among Cluster_a, Cluster_b, and Cluster_c because population pairwise Fst values among these clusters were higher than 0.25^48^. There was a greater degree of differentiation between Cluster_a and Cluster_c, with an Fst-value of 0.58246 (*P* < 0.001), than between the other pairs of clusters. The differentiation between Cluster_a and Cluster_b and that between Cluster_b and Cluster_c were similar, with Fst values of 0.46405 (*P* < 0.001) and 0.47014 (*P* < 0.001), respectively.

Geographically, we found that the original Cluster_a was distributed only among 20 populations, mainly from Southwest, North, and easternmost China. Cluster_b covered 26 populations distributed in eastern and middle portions of China. Cluster_c, the newest evolutionary clade, contained all the populations except Xiushui, Jiangxi. Most populations in western China occupied a large proportion of Cluster_c (Fig. 10b).

To assess the genetic diversity of the populations, we calculated Pi and Tajima’s *D* values based on the distribution of 132 haplotypes among 34 populations. According to the direction of *D. catenatum* migration^25^, we found that the Tajima’s *D* value of the *D. catenatum* populations decreased from west to east, regardless of the presence or absence of ancestral populations. However, an upsurge in Pi from west to east was observed if the ancestral populations were removed (Fig. 10c, d). The diversity of eastern areas was higher than that of western areas after removing the ancestral populations (ancestral populations: Cluster_a/Cluster_abc ≥ 15%). The Fst of the 34 populations ranged from 0~0.78766. Details of the molecular diversity, population pairwise Fst values, nucleotide diversity, and haplotype kinds and numbers are shown in Tables S6–8.

## Discussion

In the present study, 82 *UGT* genes were identified in *D. catenatum* and clustered into 15 groups based on phylogenetic analysis. *UGT* gene products account for ~0.28% of the gene products in *D. catenatum*, <0.44% in *A. thaliana*, 0.36% in cotton, 0.4% in chickpea, and >0.23% in maize^17,18,21,49^. In plants, 12 groups (A–G, I–M) are highly conserved, with the exception of the H and N groups^18^. Thus far, the absence of group N has been discovered in 22 species, only one of which is a monocot^41^. The loss of groups N and H during evolution suggests that either the functions of these groups are not important or these functions can be replaced by other factors^19^. Groups A, D, E, G, and L, which expanded more than others during the evolution of higher plants^50^, gained the highest, second highest, and third highest numbers (21 (D), 10 (E), and 8 (A, G, L)) members, accounting for 25%, 12%, and 9.7% of the putative *UGT*s in *D. catenatum*, respectively. Members of these groups have been reported to catalyze a wide variety of substrates, such as flavonoids, terpenes, and alkaloids^15,41^, suggesting that *D. catenatum* is rich in these kinds of metabolites. Three groups, O, P, and Q, which are found in maize but not *A. thaliana*, were also identified in *D. catenatum*. In accordance with a previous study, we concluded that group O in *D. catenatum* contained five closely related members, including four *ZOG* genes^50^. Members of group O may therefore catalyze the glycosylation of cytokinins^18^. Group Q, a group absent in Poales and Brassicales^41^, was also identified during our analysis. A novel group, R, which contains members of the UGT708 family, such as *ZmCGT*, was found in four members of *D. catenatum*. This suggests that there may be similar functional genes in *D. catenatum*.

Among the 82 genes that we identified, 78 *UGTs* were dispersed throughout the chromosomes. These *UGTs* were generally organized into clusters of 2–7 genes, and most ORFs in the clusters were oriented in the same direction. Genes in the same cluster showed high sequence similarity, reflecting their close phylogenetic relationships in the family and tandem duplication after orchid-wide genome duplication. This phenomenon also occurs in the GT1 family of cotton^21^. The position, phase, loss, and gain of introns can serve as important indicators for understanding the evolution of a gene family within phylogenetic groups. More than half (52%) of *UGTs* lack introns, which is less than the number in maize (60%) and Arabidopsis (58%). Introns were abundantly present in phase 0, suggesting that their phases remained stable. We also used the MEME web server to search the conserved motifs shared among UGT proteins. We found a total of 10 distinct conserved motifs, among which motif 1, which encoded the UGT domain, was found in all the identified UGTs. From similar motif patterns, we concluded that differences among the groups were not significant and that the UGTs in *D. catenatum* are still undergoing evolution. Although most closely related members usually shared common motifs, some UGTs, such as Dca003346, Dca009408, and Dca014393, differed from the other members in their group. We speculated that the structural annotation of genes was incomplete. The specific motifs may contribute to the functional divergence of UGTs. Gene expression patterns can help us understand the function of these genes in different tissues. Thirty-five *UGT* genes were expressed among the nine tissues evaluated in this study. Active involvement of the abovementioned groups in each organ confirms that these groups have vital roles in growth hormone glycosylation. Groups D and E not only gained the most *UGT* members in *D. catenatum* but also possessed the most genes simultaneously expressed in all organs. Comparison of the day roots and night roots indicated that 12 *UGTs* were expressed either only during the day or only at night. This result suggests that some *UGTs* are expressed at specific times.

In recent years, genome-wide identification of gene families has been used to reveal pathways used to synthesize bioactive components such as geranyl and neryl glucoside in grape^51^ and 2-pheylethanol in peach^22^. In the present study, we compared the 82 Dca UGTs with identified CGTs using multiple sequence alignment and phylogenetic/evolutionary analysis. Putative genes, grouped into a clade with identified CGTs, were chosen as candidate genes for further verification. Based on enzyme activity, a putative di-*C*-glucosylflavone pathway in *D. catenatum* was proposed. Our results show that DcaCGT catalyzed both the first and second *C*-glycosylation reactions of di-*C*-glucosylflavone. This is the second enzyme possessing this catalytic ability, in addition to FcCGT^38^. DcaCGT showed strong activity when using phloretin as the substrate and weaker activity when using 2-hydroxynaringenin as the substrate in vitro. When we used apigenin as a substrate, we found that a small amount of apigenin-7-*O*-glucoside remained in the reaction product. We concluded that DcaCGT not only catalyzed di-*C*-glucosylflavone but also processed partial active sites on *O*-glucosylflavone. However, we could not detect any *O*-glucosylflavone when we used phloretin or 2-hydroxynaringenin as a substrate. *C*-glucosylflavone was also not detected when using apigenin as a substrate. Thus, we deduced that DcaCGT exhibits high substrate specificity.

In our phytochemical analysis, the substrates 2-hydroxynaringenin, phloretin, and apigenin were detected only in the root, stem, and leaf, in which transcript expression was lower than that in the flowers and flower buds. The levels of the mono-*C*-glucosylflavonoid, di-*C*-glucosylflavonoid, and *O*-glucosylfavonoid products were higher in flower buds than in roots. Thus, we concluded that lower transcription led to a lower product content and that DcaCGT showed strong catalytic capacity in vivo. Comparing the amounts of the three products, we found that the accumulation of vicenin-2 and of its intermediate products vitexin and isovitexin was higher than that of nothofagin and cosmosiin in different tissues of *D. catenatum*. These results indicate that DcaCGT has a pivotal role in the accumulation of vicenin-2.

Flavonoids are used by organisms in response to nearly all abiotic stresses^52,53^. CGT, one of the key enzymes in the flavonoid biosynthesis pathway, has continued to expand during the evolution of *D. catenatum*. Our results show that Cluster_b/c demonstrated high differentiation resulting from ancestral and selection pressure, generating adaptable genotypes after *D. catenatum* migration. Based on the wide distribution of Cluster_c, we concluded that Cluster_c was the best genotype for acclimation. Interestingly, we found that *DcaCGT* was grouped into Cluster_c. Previous studies have reported that the accumulation of cosmosiin, which is a product of DcaCGT *O*-glycosylation, can strengthen the ability of *D. catenatum* to resist drought stress^54,55^. Most wild *D. catenatum* grows on cliffs with deficient water year round^26^, which may be the reason for the remaining *O*-glycosylation activity of DcaCGT. The distribution of Cluster_b led us to hypothesize that this genotype can better adapt to the environment of eastern and central China. However, whether CGTs in Cluster_b gained the same or other activities requires further study. Our results from population diversity analysis of *D. catenatum* migration show that populations in eastern China possess increased genetic variation. The ancestral Cluster_a can still be found in Yunnan, Guangdong, Zhejiang, and Hubei provinces. Yunnan and Guangdong are the ancestral region of *D. catenatum*^25^, which is why it shows increased diversity. We concluded that human activity may lead to increased diversity in eastern areas of China. In addition, flavonoid contents in different *D. catenatum* organs indicate that multiple haplotypes may be one of the reasons for the different levels of vicenin-2 observed in different regions.

As the functions of plant CGTs have been increasingly identified, the reaction mechanisms of plant CGTs have also been gradually elucidated. Hirade et al.^36^ showed that substituting His 20 with alanine in UGT708D1 changes *C*-glucosyltransferase activity to that of *O*-glucosyltransferase; both D85 and R292 are active sites in *C*-glucosylation. Replacing either of these sites with alanine in UGT708D1 induces the loss of *C*-glucosylation. He et al.^39^ concluded that I94 and G284 are the two active sites for *C*-glucosylation. When I94 is replaced with glutamic acid or G284 is replaced with lysine, TcCGT changes from *C*- to *O*-glucosylation. Our haplotype analysis of DcaCGT from 34 populations indicated that the active sites of His and Asp among the 274 samples examined in this study were identical. However, the active site of lle was replaced by Met, and the active site of Gly was replaced by Arg. Such changes in active sites used for *C*-glucosylation may be the reason for *O*-glucosyltransferase activity. Further studies are needed to delineate the CGT reactions in detail.

In summary, we isolated a novel di-*C-*/*O*-glycosyltransferase from the *D. catenatum* GT1 family and analyzed the levels of related components in different tissues of *D. catenatum*. The results of transcriptomic and metabolic profiling demonstrated that the flavonoid distribution differed among organs. We further examined the origin and differentiation of *DcaCGT* and concluded that the remaining OGT activity may help *D. catenatum* adapt to water deficit stress. Based on previously identified active sites on UGT708D1 and TcCGT, we found that CGT active sites were altered in *D. catenatum*. However, we did not find distinct differences between the active sites of Cluster_b and Cluster_c. Our results show that changes in these active sites can produce OGT activity. However, further studies are needed to determine whether there are functional differences between Cluster_b and Cluster_c. Our study provides a basis for understanding the impact of environmental conditions on the biosynthesis of medically relevant compounds in *D. catenatum*. These results will lay the basis for enhancing vicenin-2 production using genetic strategies and molecular breeding in *D. catenatum*.

## Conclusion

We performed a genome-wide analysis and functional verification of CGT genes in *D. catenatum*. The haplotype distribution of *DcaCGT* was assessed with 274 samples acquired from 34 populations. We isolated a novel *C*-glycosyltransferase (*DcaCGT*) from *D. catenatum* by identifying and analyzing 82 putative genes in the GT1 family. DcaCGT could specifically catalyze not only di*-C*-glycosylation but also *O-*glycosylation. Apart from the normal function of catalyzing 2-hydroxynaringenin and phloretin to the respective di-*C*-glycosides, DcaCGT also catalyzes apigenin to cosmosiin. We also established that *DcaCGT* functions expanded throughout the evolution of *D. catenatum*. The residual OGT activity may help *D. catenatum* resist drought stress. Our results reveal the biosynthetic pathways of di-*C/O*-glucosylflavones in medicinal orchids, and our molecular phylogenetic analysis offers insight into the evolution of the functional gene.

## Materials and methods

### Plant materials and chemicals

*D. catenatum* was obtained from Qujing, Yunnan, and authenticated by Professor Zhongjian Liu (College of Landscape Architecture, Fujian Agriculture and Forestry University). Flower buds, flowers, leaves, stems, and roots, collected from healthy 2-year-old *D. catenatum* plants, were frozen at −80 °C.

2-Hydroxynaringenin, vitexin, isovitexin, phloretin, apigenin, and cosmosiin were purchased from ChemFaces (Wuhan, China). UDP-glucose was purchased from Energy Chemical (Shanghai, China). Vicenin-2 was isolated from *D. catenatum* leaves in our laboratory. Nothofagin was isolated from *Desmodium caudatum* in Haifeng Chen’s laboratory (College of Pharmacy, Xiamen University).

### Identification of *UGT* genes

To identify UGTs, a feature sequence of UGTs, the 44-amino-acid conserved sequence of the PSPG motif, was used as a query to perform a local BLASTP search against the *D. catenatum* genome database. The coding sequence and chromosome distribution were obtained from the same database. The molecular weight (MW) and isoelectric point (pI) of each UGT protein were calculated using the online ExPASy (http://web.expasy.org/compute_pi/) program. The domain of each UGT protein was obtained using an NCBI batch CD search (https://www.ncbi.nlm.nih.gov/cdd/). KofamKOALA (https://www.genome.jp/tools/kofamkoala/) was used to confirm the function of UDP-glycosyltransferase among the UGT candidates. The subcellular localization of each UGT protein was predicted using the online CELLO v2.5 system (http://cello.life.nctu.edu.tw/). The distribution of *UGT* genes and their location on chromosomes were visualized using TB tools (https://omictools.com/tbtools-tool).

### Sequence alignment and phylogenetic analysis

The predicted amino-acid sequences of *D. catenatum* UGTs were aligned by the neighbor-joining method in ClustalX software using 10,000 bootstrap replicates. A phylogenetic tree was constructed using FigTree 1.4.3.

### Sequence analysis

The exon–intron organization of *D. catenatum UGTs* was evaluated by determining intron lengths, positions, and phases in the genome. Intron phases were determined as follows: introns positioned between two triplet codons were defined as phase 0; introns positioned after the first and second base in the codon were defined as phases 1 and 2, respectively. Conserved motifs were analyzed using the MEME online program (http://meme.nbcr.net/meme/intro.html).

### Gene expression analysis using RNA-seq

Transcriptomic data available for putative *UGT* expression in different plant organs were analyzed according to the data published by Zhang et al.^29^. Samples of each organ (flower bud, gynandrium, labellum, sepal, root tip, root, leaf, stem, and pollen), obtained from three or more different plants, were pooled prior to sequencing. Gene expression levels are shown as FPKM values. Transcript profiles for selected *D. catenatum UGTs* were expressed as heatmaps.

### Cloning of *DcaCGT* and production of recombinant protein

Total RNA of *D. catenatum* was extracted from frozen leaves using a Quick RNA Isolation Kit per the manufacturer’s instructions (Huayueyang, Beijing, China). First-strand cDNA was synthesized from total RNA (568 ng) using TransScript All-in-One First-Strand cDNA Synthesis SuperMix for PCR (TransGen Biotech, Beijing, China). PCR was performed using 1 μL first-strand cDNA as a template and *DcaCGT*-F and *DcaCGT*-R as primers (the primer sequences are provided in Table S3). The coding sequence (CDS) of the candidate CGT gene, *DcaCGT*, was amplified using PrimerSTAR Max DNA polymerase (Takara Bio, Japan) under the following conditions: 94 °C for 5 min, 37 cycles of 94 °C for 30 sec, 55 °C for 30 sec, and 72 °C for 90 sec, followed by 72 °C for 10 min. The amplified fragment was purified from an agarose gel (Magen, China), cloned using pEAST-T5 vector (TransGen Biotech, Beijing, China), transformed in phage-resistant chemically competent *E. coli* cells (TransGen Biotech, Beijing, China), and sequenced.

The empty vector pET32a (+) (Novagen, USA) was digested with FastDigest enzymes Sca I and EcoR V (Thermo, USA). Full-length cDNA was amplified by PCR using pETF-*DcaCGT* and pETR-*DcaCGT* as primers (Table S3) and purified from an agarose gel. To obtain the recombinant plasmid, the target gene was linked with a linearized vector using an Infusion cloning system (Clontech, Japan) and transformed into *E. coli* Transetta (DE3) (TransGen Biotech, Beijing, China); 0.01 mm isopropyl β- d-1-thiogalactopyranoside was then added, and the cells were incubated for 20 h at 16 °C. The cell cultures were centrifuged at 6574 × *g*, after which the supernatants were removed and harvested *E. coli* cells were stored at −20 °C. The *E. coli* cells were suspended in 1 × PBS (containing 1 mm PMSF and 20 mm imidazole) and disrupted by sonication. After centrifugation at 15,000 × *g* for 30 min at 4 °C, the supernatants were applied to a Ni-NTA Superflow column (Qiagen, Germany) with a linear gradient of imidazole, followed by desalination-exchange buffer (pH 8.0) containing 1 mm DTT on a PD-10 Column (GE Healthcare).

### UGT enzymatic activity assay

Enzymatic reactions were performed at 30 °C for 5–90 min using a 250 μm acceptor substrate, UDP-glucoside, 1 mm DTT, 50 mm Tris-HCl (pH 8.0), and 300 ng purified protein, resulting in a total volume of 100 µl. The reaction was terminated by adding 10 μL 2 m HCl, followed by incubation at 60 °C for 30 min to dehydration. The protein was boiled at 100 °C for 10 min as a negative control.

Supernatants were dissolved in 100 µl HPLC-grade methanol and analyzed via HPLC-MS/MS on an AB Sciex Triple-TOF 5600^+^ mass spectrometer coupled to a Shimadzu LC-30 AD chromatography system. Products were detected by measuring absorbance at 280 nm and 340 nm.

### HPLC-MS analysis

Flower buds, flowers, leaves, stems, and roots of *D. catenatum* were ground into a powder in liquid nitrogen. In total, 100 mg powder was weighed and transferred into a conical flask. Then, 40 mL methanol was added, and the mixture was extracted ultrasonically for 1 h at 30 °C. Extracts were concentrated using a hypobaric drying method at 60 °C. Then, 5 mL HPLC-grade methanol was used to redissolve the residues to obtain the extraction solution. All the samples were filtered through a 0.22-μm membrane filter before analysis via HPLC-MS. 2-Hydroxynaringenin, vitexin, isovitexin, vicenin-2, phloretin, nothofagin, apigenin, and cosmosiin were confirmed by HPLC-MS analysis using authentic standards. 3’,5’-Di-*C*-glucosylphloretin was identified by the MS^2^ fragment. Loratadine was chosen as the internal standard. The quantification of these flavonoids was performed based on their corresponding standard curves. Each tissue type was analyzed in three biological replicates in this experiment.

HPLC-MS analysis was performed with UPLC-MS/MS (AB Sciex Triple-TOF 5600^+^ mass spectrometer coupled to a Shimadzu LC-30 AD chromatography system) to complete qualitative analysis. The detected components were sent to a Thermo Fisher UHPLC-MS/MS system consisting of an Accela ultra-high-pressure liquid chromatograph and a TSQ Quantum Ultra triple-quadrupole mass spectrometer (Thermo Fisher Scientific, San Jose, CA, USA) fitted with an ESI source for quantitative analysis. All separations were carried out with an SB-C18 column (2.1 × 100 mm, 1.8 μm; Agilent). The system was operated in negative ion mode at a flow rate of 0.3 mL/min using solvent A (water with 0.1% formic acid), solvent B (acetonitrile), and C (methanol). The gradient elution started from 7% B, 7% C for 0 min, followed by 9% B, 9% C in 0.3 min; 11% B, 11% C in 2.7 min; 13% B, 13% C in 3.8 min; 14% B, 14% C in 4.8 min; 18% B, 18% C in 6.5 min; 45% B, 45% C in 8.5 min; a hold for 0.7 min; and a post run time of 1.5 min for re-equilibration. An optimal MS signal response of eight analytes and IS was obtained with the following ion source parameters: spray voltage at 3500 V, capillary temperature at 320 °C, sheath gas pressure at 35 psi, and auxiliary gas pressure at 10 psi.

### Haplotype analysis of *DcaCGT*

A total of 274 samples of *D. catenatum* from 34 populations and five samples of *D. huoshanense* from one population were newly resequenced using 150 paired-end cycles on a NovaSeq 6000 instrument. After quality trimming, paired-end reads were mapped to the *D. catenatum* reference genome using BWA-MEM v0.7.12-r1039 with default parameters^56^. The SortSam and MarkDuplicates commands from Picard Tools v1.56 were used to mark and remove duplicates in the bam-format files. IndelRealigner and RealignerTargetCreator from GenomeAnalysisTK v3.8–1 were used to perform local realignment^57^. For each sample, individual variant calling was conducted using Haplotyper and GVCFtyper from Sentieon v201711.03^58^. Coding sequences for *DcaCGT* from 274 samples were obtained using the faidx tool from SAMtools v1.9 and consensus from BCFtools v1.2^59^.

The 274 *DcaCGT* sequences were aligned by ClustalX software in MEGA5. A phylogenetic tree was constructed using the *DcaCGT*-coding sequences and neighbor-joining method in MEGA5. Population genetic analysis was performed by using DnaSP 6^60^. Nei’s nucleotide diversity (*π*) and Fst were computed by Arlequin v.3.0^61^.

## Supplementary information

Supplemental material
