# Supplementary material for: Functional analysis of a novel C-glycosyltransferase in the orchid Dendrobium catenatum
Source: Hortic Res. 2020 Jul 1;7:111. doi: 10.1038/s41438-020-0330-4 (PMC7326982; doi:10.1038/s41438-020-0330-4)
Supplement: Supplementary file 1 — Supplemental material [file 41438_2020_330_MOESM1_ESM.docx]

**
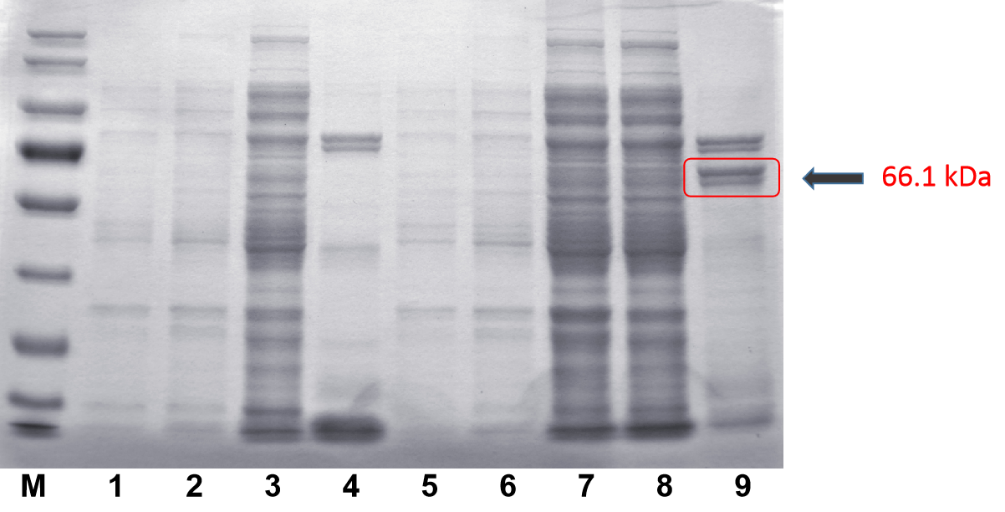
**

**Figure S1.** SDS-PAGE analysis of DcaCGT protein expression and purification. 1-4 represent pET32a (+) empty plasmid; 5-9 represent pET32a (+) with target gene. M, Marker; 1 and 5, IPTG (-); 2 and 6, IPTG (+) without ultrasonic grinder; 3,7 and 8, Crude enzyme; 4, Purified empty plasmid; 8 Purified DcaCGT fused with His-tag (predicted molecular weight:66.1 kDa).


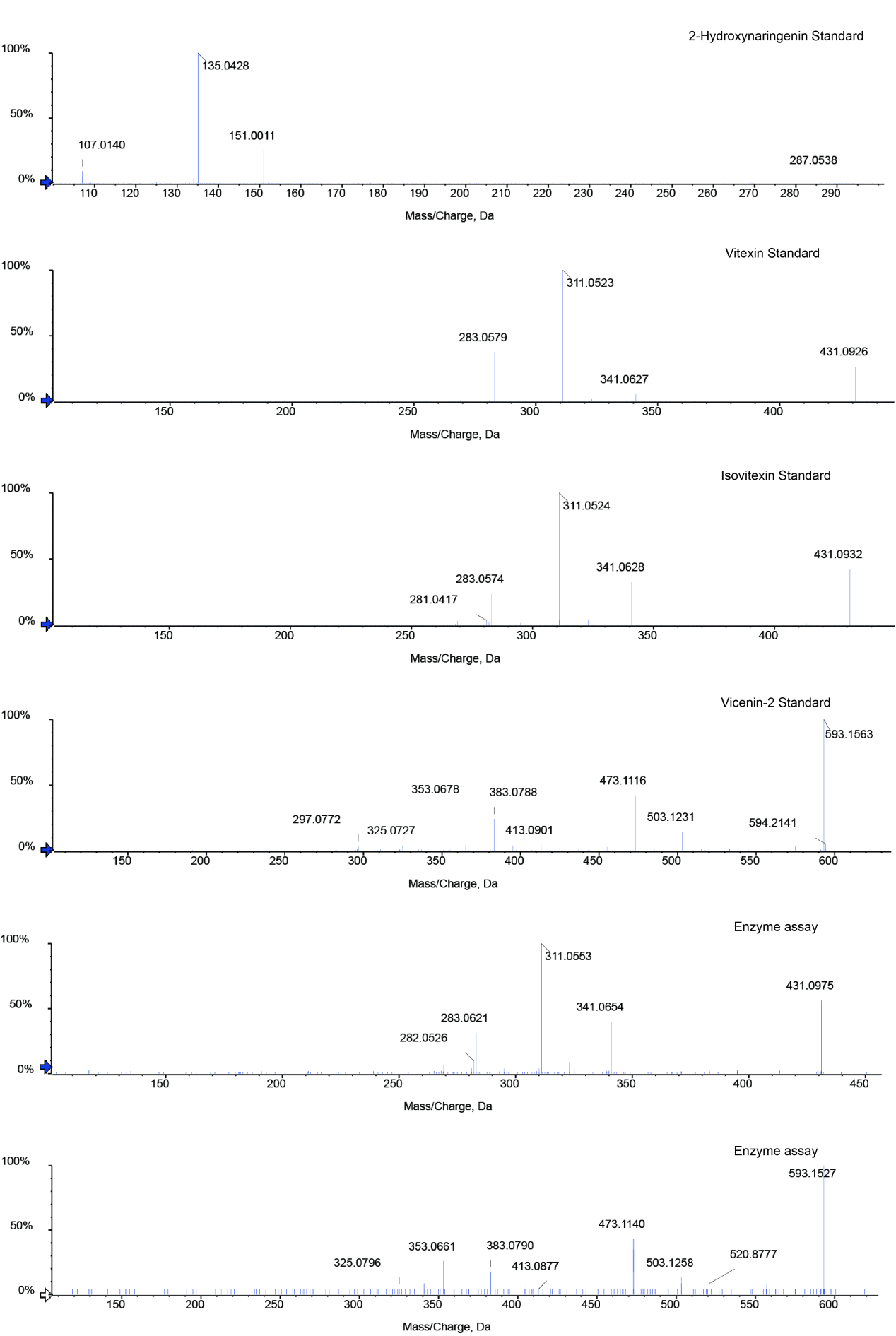


**Figure S2.** The MS/MS fragmentation profile for 2-Hydroxynaringenin/ Vitexin/ Isovitexin/ Vicenin-2 standard and the corresponding product of enzyme assay.


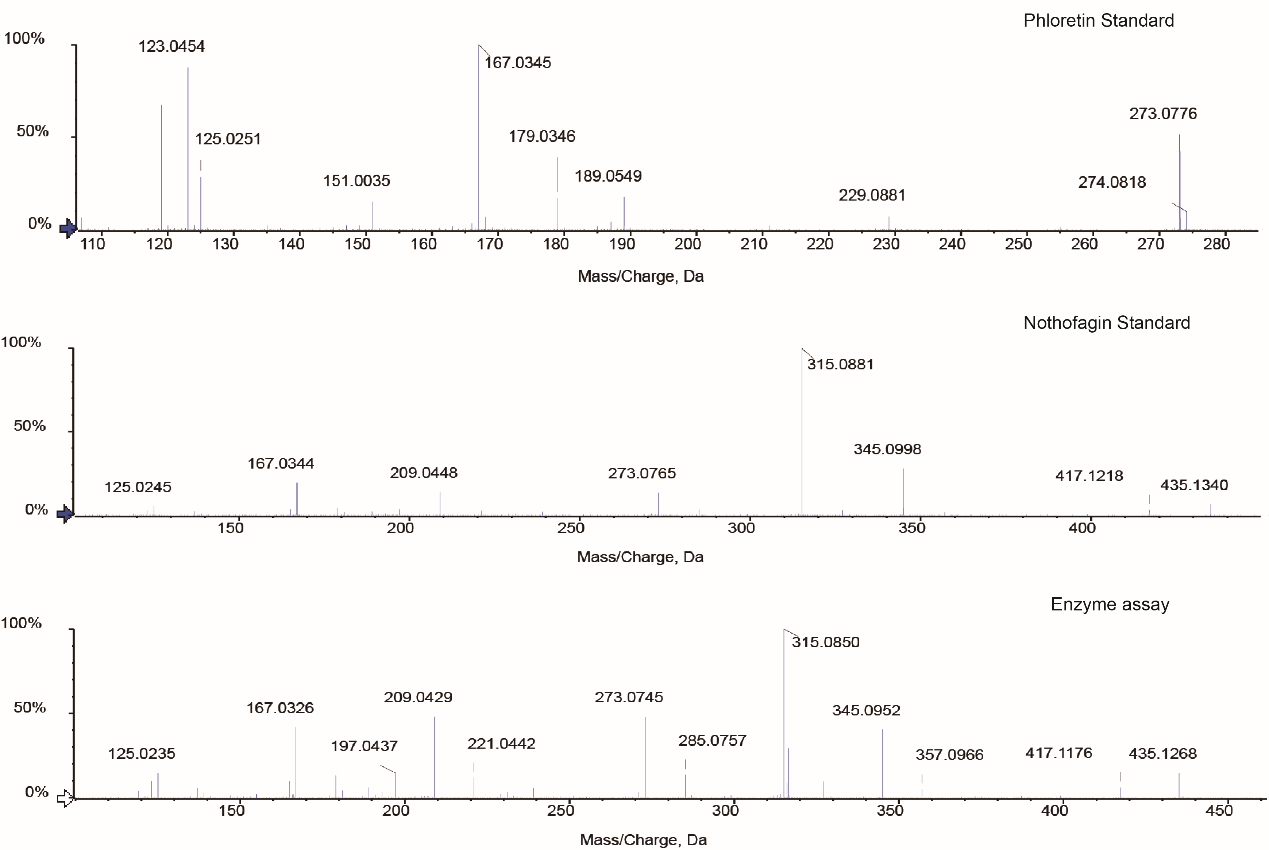


**Figure S3.** The MS/MS fragmentation profile for Phloretin/ Nothofagin standard and the corresponding product of enzyme assay.


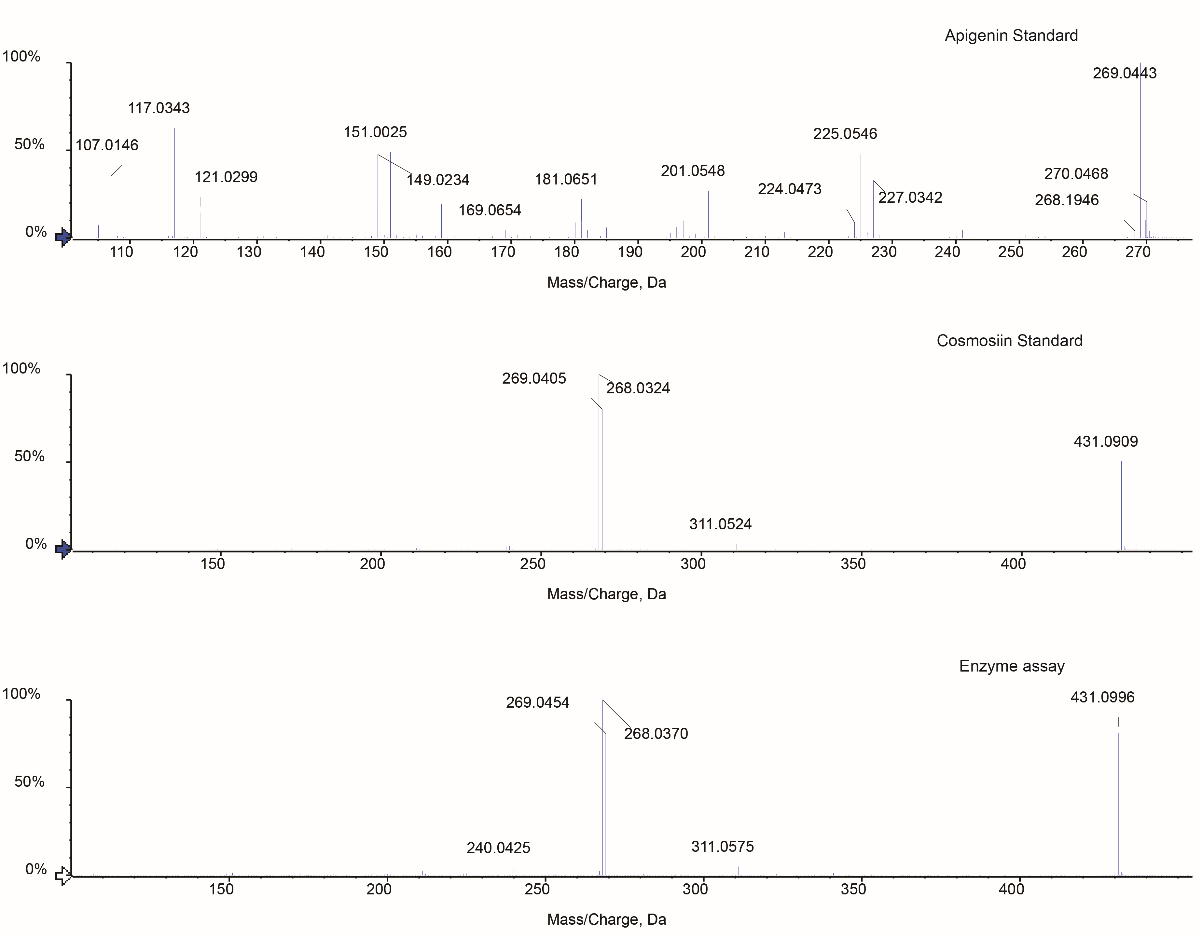


**Figure S4.** The MS/MS fragmentation profile for Apigenin/ Cosmosiin standard and the corresponding product of enzyme assay.


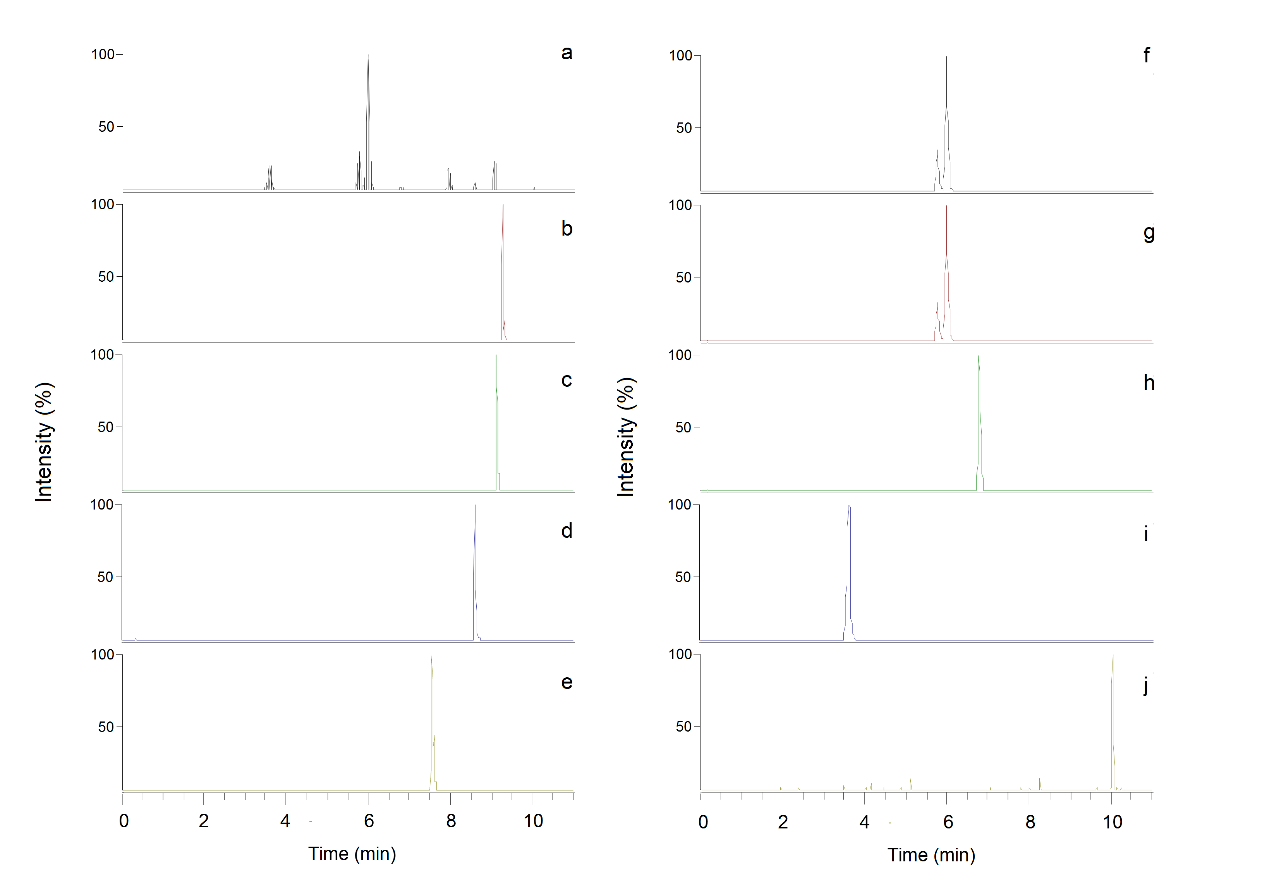


**Figure S5.** UHPLC-TQ-MS/MS analysis SRM chromatogram of 8 analytes and internal standard (IS). (a) TIC; (b) Phloretin; (c) 2-Hydroxynaringenin; (d) Apigenin; (e) Cosmosiin; (f) Vitexin; (g) Isovitexin; (h) Nothofagin; (i) Vicenin-2; (j) IS.


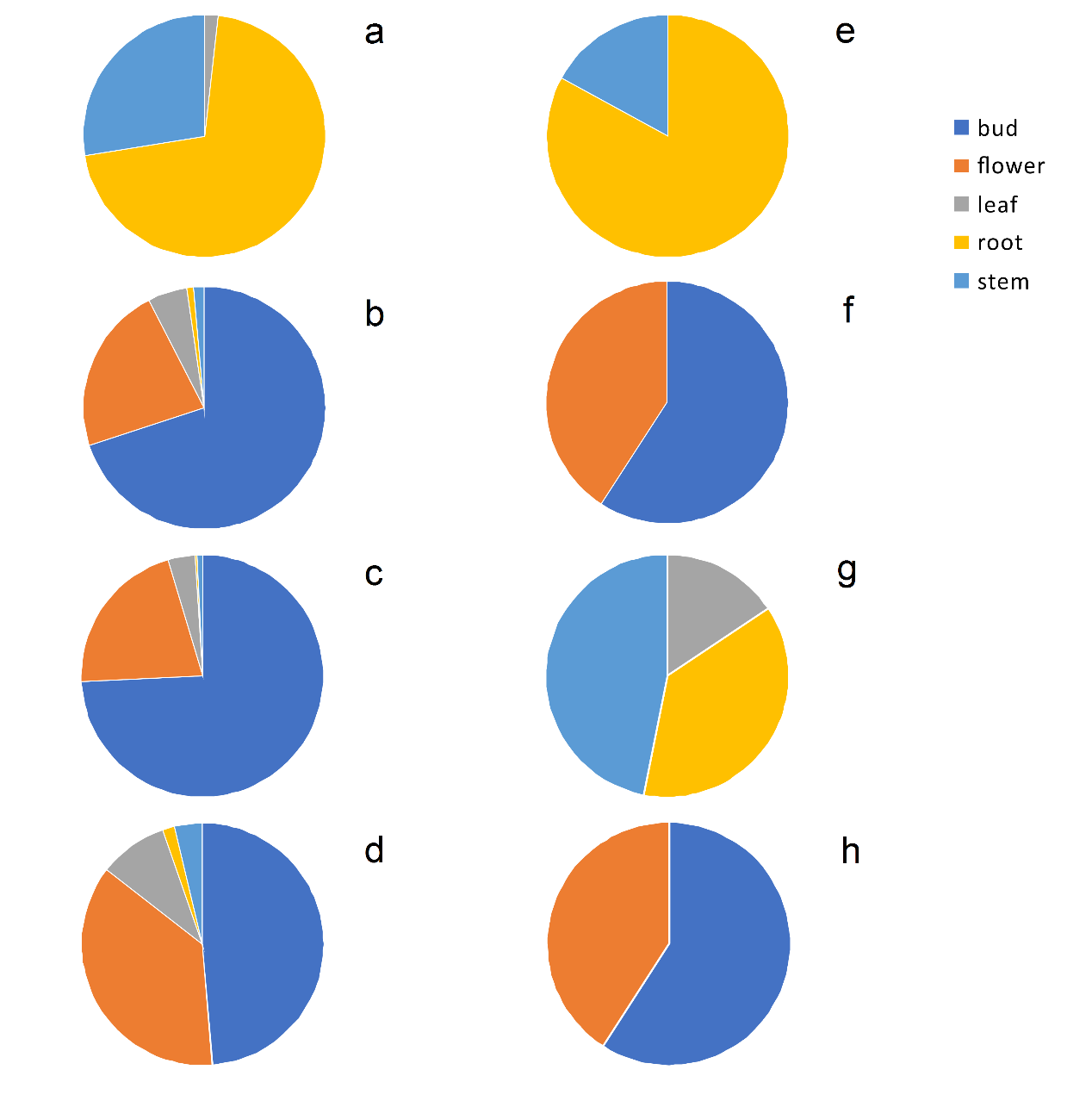


**Figure S6.** Percentage of (a) 2-Hydroxynaringenin; (b) Vitexin; (c) Isovitexin; (d) Vicenin-2; (e) Phloretin; (f) Nothofagin; (g) Apigenin; (h) Cosmosiin in different tissues of *D. catenatum.*


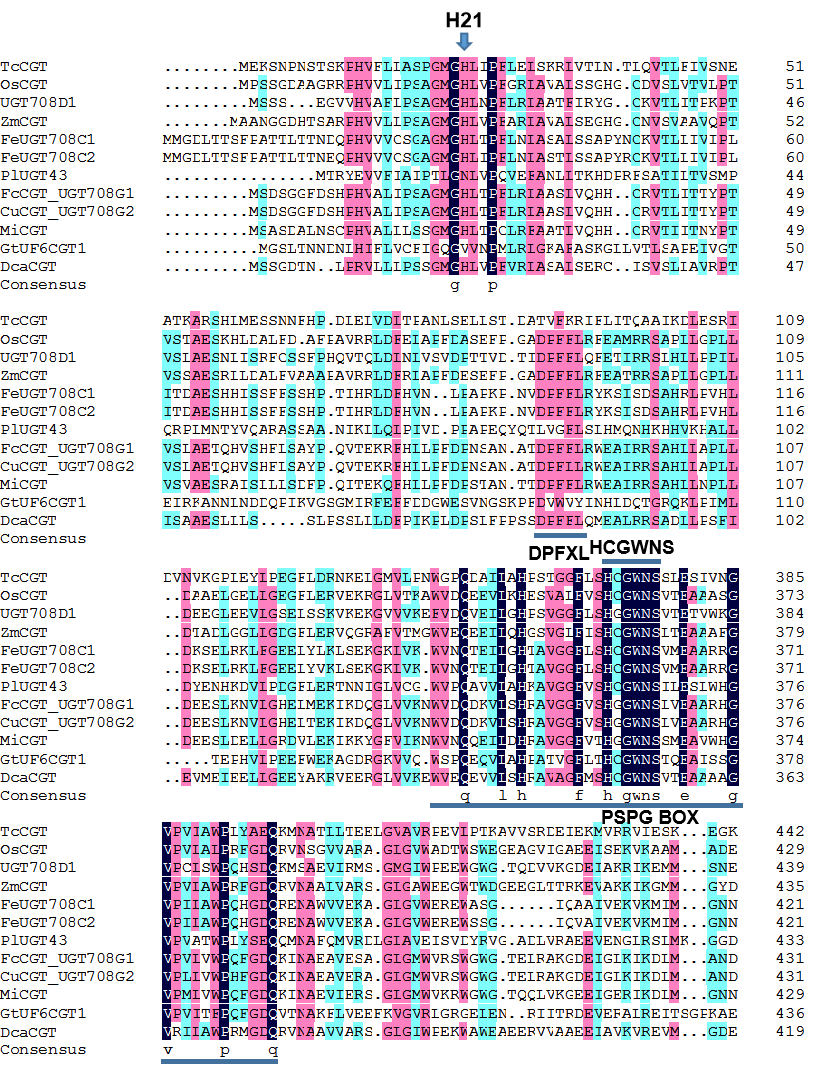


**Figure S7.** Alignment of the amino acid sequences of DcaCGT and other plant *C*-glycosyltransferases. Amino acid sequences were aligned by DNAMAN 8.0. The blue lines represented the conserved motif of UGTs (PSPG BOX) and the substrates characteristic motif of plant CGTs (DPFXL).

**Table S1.** Information of the 82 putative UGTs in *Dendrobium catenatum*.

| Group | Gene Name | pI | MW  (Da) | Size  (aa) | ORF length  (bp) | Predicted Subcellular location | Chr.  No. | NCBI CD-Search (Domain) | | | KO |
| --- | --- | --- | --- | --- | --- | --- | --- | --- | --- | --- | --- |
|  |  |  |  |  |  |  |  | From | to | Short name |  |
| B | Dca000014 | 5.95 | 48949.29 | 458 | 1377 | InnerMembrane | 1 | 19 | 449 | Glycosyltransferase_GTB-type |  |
| D | Dca000263 | 5.66 | 50831.47 | 461 | 1386 | InnerMembrane | 1 | 12 | 457 | Glycosyltransferase_GTB-type |  |
| I | Dca000855 | 5.75 | 54325.83 | 486 | 1461 | InnerMembrane | 1 | 12 | 483 | Glycosyltransferase_GTB-type |  |
| O | Dca001361 | 6.23 | 51415.41 | 465 | 1398 | Cytoplasmic | 7 | 16 | 455 | GT1_Gtf-like | K13495 |
| D | Dca002345 | 5.24 | 51647.14 | 474 | 1425 | Cytoplasmic | 2 | 7 | 455 | Glycosyltransferase_GTB-type |  |
| L | Dca002610 | 5.36 | 48514.19 | 443 | 1332 | Cytoplasmic | 2 | 5 | 441 | Glycosyltransferase_GTB-type |  |
| B | Dca003244 | 5.92 | 51549.4 | 474 | 1425 | InnerMembrane | 9 | 1 | 453 | Glycosyltransferase_GTB-type |  |
| C | Dca003346 | 4.79 | 38403.86 | 348 | 1047 | Cytoplasmic | 9 | 20 | 311 | Glycosyltransferase_GTB-type |  |
| G | Dca003571 | 5.37 | 53215.73 | 478 | 1437 | Cytoplasmic | 4 | 9 | 474 | Glycosyltransferase_GTB-type |  |
| E | Dca004262 | 6.45 | 49952.8 | 461 | 1386 | Cytoplasmic | 4 | 7 | 417 | Glycosyltransferase_GTB-type |  |
| A | Dca004914 | 5.25 | 50078.26 | 445 | 1338 | Cytoplasmic | 14 | 1 | 439 | Glycosyltransferase_GTB-type |  |
| A | Dca004915 | 5.23 | 50114.36 | 446 | 1341 | Cytoplasmic | 14 | 3 | 440 | Glycosyltransferase_GTB-type |  |
| A | Dca004917 | 5.3 | 51720.25 | 462 | 1389 | Cytoplasmic | 14 | 17 | 456 | Glycosyltransferase_GTB-type |  |
| D | Dca006328 | 7.13 | 45216.4 | 404 | 1215 | Cytoplasmic | 1 | 1 | 396 | Glycosyltransferase_GTB-type |  |
| D | Dca006464 | 5.75 | 55190.69 | 496 | 1491 | Cytoplasmic | 2 | 14 | 489 | Glycosyltransferase_GTB-type | K13496 |
| O | Dca007365 | 6.6 | 52561.06 | 472 | 1419 | Cytoplasmic | 2 | 214 | 472 | Glycosyltransferase_GTB-type | K13495 |
| O | Dca007367 | 6.02 | 50204.87 | 457 | 1374 | InnerMembrane | 2 | 198 | 457 | Glycosyltransferase_GTB-type | K13495 |
| D | Dca007531 | 6.02 | 55402.93 | 499 | 1500 | Cytoplasmic | 1 | 17 | 489 | Glycosyltransferase_GTB-type |  |
| D | Dca007533 | 5.91 | 54755.19 | 496 | 1491 | Cytoplasmic | 1 | 15 | 486 | Glycosyltransferase_GTB-type |  |
| D | Dca007536 | 6.46 | 55864.87 | 502 | 1509 | Cytoplasmic | 1 | 9 | 489 | Glycosyltransferase_GTB-type |  |
| O | Dca007566 | 7.76 | 49060.42 | 440 | 1323 | Cytoplasmic | 1 | 192 | 435 | Glycosyltransferase_GTB-type | K13495 |
| G | Dca008131 | 5.38 | 56981.73 | 513 | 1542 | Cytoplasmic | 2 | 33 | 508 | Glycosyltransferase_GTB-type |  |
| G | Dca008134 | 4.99 | 53455.32 | 486 | 1461 | Cytoplasmic | 2 | 10 | 482 | Glycosyltransferase_GTB-type |  |
| G | Dca008135 | 5.66 | 52980.07 | 479 | 1440 | Cytoplasmic | 2 | 9 | 478 | Glycosyltransferase_GTB-type |  |
| G | Dca008136 | 5.45 | 53109.98 | 479 | 1440 | Cytoplasmic | 2 | 9 | 479 | Glycosyltransferase_GTB-type |  |
| D | Dca008182 | 5.27 | 54090.97 | 487 | 1464 | Cytoplasmic | 2 | 8 | 476 | Glycosyltransferase_GTB-type | K23260 |
| D | Dca008184 | 5.79 | 53323.47 | 481 | 1446 | Cytoplasmic | 2 | 6 | 477 | Glycosyltransferase_GTB-type | K23260 |
| L | Dca008211 | 5.12 | 52313.74 | 479 | 1440 | Cytoplasmic | 3 | 1 | 450 | Glycosyltransferase_GTB-type |  |
| R | Dca008486 | 5.32 | 48609.33 | 453 | 1362 | InnerMembrane | 1 | 9 | 441 | Glycosyltransferase_GTB-type | K23154 |
| R | Dca008487 | 5.97 | 48834.55 | 453 | 1362 | InnerMembrane | 1 | 11 | 449 | Glycosyltransferase_GTB-type | K23154 |
| R | Dca008548 | 5.4 | 48730.56 | 453 | 1362 | InnerMembrane | 1 | 11 | 449 | Glycosyltransferase_GTB-type | K23154 |
| O | Dca009088 | 5.82 | 51027.25 | 474 | 1425 | Cytoplasmic | 5 | 2 | 441 | Glycosyltransferase_GTB-type |  |
| P | Dca009377 | 5.14 | 54416.36 | 488 | 1467 | Cytoplasmic | 16 | 14 | 476 | Glycosyltransferase_GTB-type |  |
| P | Dca009379 | 5.61 | 53563.42 | 483 | 1452 | Cytoplasmic | 16 | 5 | 476 | Glycosyltransferase_GTB-type |  |
| A | Dca009408 | 5.82 | 36872.61 | 331 | 996 | Cytoplasmic | 16 | 1 | 300 | GT1_Gtf-like |  |
| F | Dca009489 | 5.83 | 39862.1 | 368 | 1107 | Cytoplasmic | 1 | 26 | 343 | GT1_Gtf-like | K12930 |
| L | Dca009739 | 5.45 | 52731.83 | 477 | 1434 | Cytoplasmic | 4 | 12 | 477 | Glycosyltransferase_GTB-type |  |
| E | Dca010206 | 6.12 | 51595.54 | 472 | 1419 | Cytoplasmic | 10 | 7 | 469 | Glycosyltransferase_GTB-type | K08237 |
| D | Dca011222 | 6.15 | 55766.56 | 499 | 1500 | Cytoplasmic | 3 | 13 | 494 | Glycosyltransferase_GTB-type | K13496 |
| J | Dca011372 | 5.05 | 51456.15 | 469 | 1410 | Cytoplasmic | 11 | 6 | 462 | Glycosyltransferase_GTB-type |  |
| A | Dca011900 | 5.33 | 50862.5 | 462 | 1389 | Cytoplasmic | 3 | 6 | 453 | Glycosyltransferase_GTB-type |  |
| A | Dca011901 | 6.69 | 55169.36 | 498 | 1497 | InnerMembrane | 3 | 46 | 489 | Glycosyltransferase_GTB-type |  |
| E | Dca012026 | 6.16 | 49771.5 | 452 | 1359 | Cytoplasmic | 3 | 5 | 446 | Glycosyltransferase_GTB-type |  |
| E | Dca012110 | 5.77 | 52032.98 | 472 | 1419 | Cytoplasmic | 3 | 17 | 444 | Glycosyltransferase_GTB-type | K08237 |
| E | Dca012722 | 5.46 | 51778.38 | 467 | 1404 | Cytoplasmic | 1 | 8 | 448 | Glycosyltransferase_GTB-type | K08237 |
| E | Dca012838 | 8.61 | 52221.23 | 474 | 1425 | Periplasmic | 1 | 7 | 467 | Glycosyltransferase_GTB-type | K08237 |
| C | Dca014177 | 5.37 | 54905.28 | 502 | 1509 | Cytoplasmic | 1 | 14 | 465 | Glycosyltransferase_GTB-type |  |
| C | Dca014179 | 5.45 | 54812.19 | 502 | 1509 | Cytoplasmic | 1 | 14 | 465 | Glycosyltransferase_GTB-type |  |
| D | Dca014181 | 7.22 | 51568.81 | 472 | 1419 | Cytoplasmic | 1 | 11 | 466 | Glycosyltransferase_GTB-type |  |
| D | Dca014182 | 5.52 | 57408.14 | 512 | 1539 | Cytoplasmic | 1 | 10 | 502 | Glycosyltransferase_GTB-type |  |
| D | Dca014184 | 5.31 | 56022.72 | 504 | 1515 | Cytoplasmic | 1 | 10 | 502 | Glycosyltransferase_GTB-type |  |
| D | Dca014185 | 5.69 | 56602.47 | 506 | 1521 | Cytoplasmic | 1 | 10 | 487 | Glycosyltransferase_GTB-type |  |
| D | Dca014186 | 5.3 | 55997.53 | 504 | 1515 | Cytoplasmic | 1 | 10 | 500 | Glycosyltransferase_GTB-type |  |
| D | Dca014392 | 6.54 | 53396.92 | 473 | 1422 | Cytoplasmic | 4 | 9 | 466 | Glycosyltransferase_GTB-type | K23260 |
| D | Dca014393 | 6.93 | 47914.73 | 425 | 1278 | Cytoplasmic | 4 | 1 | 423 | Glycosyltransferase_GTB-type |  |
| D | Dca014394 | 5.95 | 53230.46 | 473 | 1422 | Cytoplasmic | 4 | 8 | 471 | Glycosyltransferase_GTB-type | K23260 |
| M | Dca014442 | 6.48 | 53044.2 | 477 | 1434 | Cytoplasmic | 1 | 3 | 472 | Glycosyltransferase_GTB-type |  |
| L | Dca014751 | 5.09 | 53091.36 | 484 | 1455 | Cytoplasmic | 13 | 1 | 483 | Glycosyltransferase_GTB-type |  |
| A | Dca015155 | 5.48 | 49508.98 | 463 | 1392 | Cytoplasmic | 3 | 13 | 454 | Glycosyltransferase_GTB-type | K22772 |
| A | Dca015281 | 6.15 | 51965.51 | 475 | 1428 | InnerMembrane | 8 | 6 | 408 | Glycosyltransferase_GTB-type |  |
| D | Dca015312 | 5.3 | 54263.79 | 495 | 1488 | Cytoplasmic | 8 | 19 | 482 | Glycosyltransferase_GTB-type |  |
| E | Dca015741 | 5.5 | 50936.74 | 469 | 1410 | Periplasmic | 8 | 10 | 467 | Glycosyltransferase_GTB-type | K08237 |
| E | Dca016384 | 6.36 | 50688.6 | 460 | 1383 | InnerMembrane | 1 | 10 | 454 | Glycosyltransferase_GTB-type |  |
| E | Dca018424 | 6.16 | 51645.88 | 479 | 1440 | Cytoplasmic | 6 | 4 | 471 | Glycosyltransferase_GTB-type | K22845 |
| D | Dca019011 | 5.17 | 57626.25 | 516 | 1551 | Cytoplasmic | 1 | 10 | 487 | Glycosyltransferase_GTB-type | K13496 |
| G | Dca019145 | 5.53 | 56204.53 | 497 | 1494 | Cytoplasmic | 3 | 12 | 475 | Glycosyltransferase_GTB-type |  |
| G | Dca019146 | 5.18 | 65402.14 | 578 | 1737 | Cytoplasmic | 3 | 114 | 568 | Glycosyltransferase_GTB-type | K23452/ K00699 |
| G | Dca019147 | 5.48 | 55853.98 | 506 | 1521 | Cytoplasmic | 3 | 1 | 486 | Glycosyltransferase_GTB-type |  |
| E | Dca019317 | 8.9 | 56851.5 | 519 | 1560 | InnerMembrane | 3 | 7 | 470 | Glycosyltransferase_GTB-type | K08237 |
| R | Dca019962 | 8.16 | 55405.12 | 512 | 1539 | InnerMembrane | 6 | 48 | 414 | Glycosyltransferase_GTB-type | K23154 |
| I | Dca020614 | 5.61 | 49371.94 | 435 | 1308 | Cytoplasmic | 15 | 1 | 424 | Glycosyltransferase_GTB-type |  |
| I | Dca020615 | 5.86 | 50816.82 | 448 | 1347 | Cytoplasmic | 15 | 1 | 422 | Glycosyltransferase_GTB-type |  |
| Q | Dca020691 | 5.8 | 51214.9 | 470 | 1413 | InnerMembrane | 10 | 5 | 465 | Glycosyltransferase_GTB-type |  |
| P | Dca021723 | 8.7 | 68154.25 | 609 | 1830 | OuterMembrane | 9 | 179 | 578 | GT1_Gtf-like |  |
| K | Dca022922 | 6.44 | 51666.19 | 467 | 1404 | Cytoplasmic | 5 | 7 | 460 | Glycosyltransferase_GTB-type |  |
| L | Dca024061 | 5.79 | 52588.82 | 479 | 1440 | Cytoplasmic | 4 | 13 | 479 | Glycosyltransferase_GTB-type |  |
| L | Dca024062 | 5.42 | 53491.83 | 479 | 1440 | Cytoplasmic | 4 | 13 | 479 | Glycosyltransferase_GTB-type |  |
| L | Dca024871 | 5.69 | 52890.2 | 473 | 1422 | Cytoplasmic | 1 | 16 | 471 | Glycosyltransferase_GTB-type |  |
| L | Dca026746 | 5.93 | 41502.05 | 360 | 1083 | Cytoplasmic | - | 5 | 358 | Glycosyltransferase_GTB-type |  |
| D | Dca027036 | 5.14 | 56954.73 | 511 | 1536 | Cytoplasmic | - | 11 | 510 | Glycosyltransferase_GTB-type |  |
| C | Dca028143 | 5.56 | 55682.19 | 507 | 1524 | Cytoplasmic | - | 14 | 465 | Glycosyltransferase_GTB-type |  |
| P | Dca028752 | 5.17 | 32762.31 | 297 | 894 | Cytoplasmic | - | 5 | 285 | Glycosyltransferase_GTB-type |  |

**Table S2.** 34 Referenced UGTS used to contruct phylogenetic Tree.

| **Name** | **Phylogeny** | **Species** | **Accession Number** |
| --- | --- | --- | --- |
| AtUGT79B1 | A | Arabidopsis | NP_200217.1 |
| AtUGT91A1 | A | Arabidopsis | AAD15567.1 |
| AtUGT89B1 | B | Arabidopsis | NP_177529.2 |
| AtUGT90A1 | C | Arabidopsis | NP_179281.3 |
| AtUGT73C1 | D | Arabidopsis | NP_181213.1 |
| AtUGT71B1 | E | Arabidopsis | NP_188812.1 |
| AtUGT72C1 | E | Arabidopsis | CAB16822.1 |
| AtUGT88A1 | E | Arabidopsis | NP_850597.1 |
| AtUGT78D3 | F | Arabidopsis | NP_197205.1 |
| AtUGT85A1 | G | Arabidopsis | AEE30237 |
| AtUGT76B1 | H | Arabidopsis | NP_187742.1 |
| AtUGT83A1 | I | Arabidopsis | NP_186859.1 |
| AtUGT87A1 | J | Arabidopsis | NP_180576.1 |
| AtUGT86A1 | K | Arabidopsis | NP_181234.1 |
| AtUGT74C1 | L | Arabidopsis | NP_180738.1 |
| AtUGT92A1 | M | Arabidopsis | NP_196793.1 |
| AtUGT82A1 | N | Arabidopsis | NP_188864.1 |
| GRMZM2G174192 | A | Maize | NP_001147268.2 |
| GRMZM2G091176 | C | Maize | ACL53799.1 |
| GRMZM2G179063 | D | Maize | NP_001168657.1 |
| GRMZM5G854655 | D | Maize | NP_001152201.2 |
| GRMZM2G067361 | D | Maize | NP_001168299.1 |
| ZmCGT (UGT708A6) | R | Maize | NP_001132650.2 |
| GRMZM2G049798 | G | Maize | NP_001150098.2 |
| GRMZM2G083935 | G | Maize | NP_001168449.1 |
| GRMZM2G117878 | H | Maize | NP_001146547.2 |
| GRMZM2G128504 | I | Maize | NP_001131902.2 |
| GRMZM2G417945 | L | Maize | NP_001151310.1 |
| GRMZM2G344993 | L | Maize | NP_001142122.1 |
| GRMZM2G082249 | M | Maize | NP_001147564.1 |
| GRMZM2G110816 | N | Maize | NP_001169852.1 |
| GRMZM2G168474 | O | Maize | NP_001105017.1 |
| GRMZM5G834303 | P | Maize | NP_001148991.2 |
| GRMZM2G113653 | Q | Maize | NP_001142257.1 |

**Table S3.** Specific primers for DcaCGT.

| **Gene** | **Primer sequence (5'→3')** |
| --- | --- |
| *DcaCGT*-F | ATGTCCTCAGGCGACACT |
| *DcaCGT*-R | TCACAATTTCTTTAACAAATAGCC |
| pETF-*DcaCGT* | CGACGACAAGGCCATGGCTGATATCATGTCCTCAGGCGACACTAACCTTCCT |
| pETR-*DcaCGT* | GCGGCCGCAAGCTTGTCGACGGAGCTCTCACAATTTCTTTAACAAATAGCCGCAGCTCTGATCCAAG |

**Table S4.** Mass spectrometry conditions of the 8 investigated compounds and internal standard (IS).

| **Name** | **RT (min)** | **Ionization Mode** | **Parent/Product ion** | **Collision energy(eV)** | **Tube Lens** |
| --- | --- | --- | --- | --- | --- |
| 2-Hydroxynaringenin | 8.59 | [M-H]^-^ | 286.97/151.00 | 17 | 61 |
| Vitexin | 5.81 | [M-H]^-^ | 430.95/311.00 | 23 | 83 |
| Isovitexin | 6.03 | [M-H]^-^ | 430.88/311.00 | 24 | 81 |
| Vicenin-2 | 3.64 | [M-H]^-^ | 592.99/352.90 | 37 | 109 |
| Phloretin | 9.12 | [M-H]^-^ | 273.74/167.80 | 17 | 51 |
| Nothofagin | 6.8 | [M-H]^-^ | 435.09/314.90 | 19 | 45 |
| Apigenin | 9.26 | [M-H]^-^ | 268.76/117.10 | 39 | 69 |
| Cosmosiin | 7.58 | [M-H]^-^ | 430.76/268.00 | 35 | 95 |
| Loratadine (IS) | 10.03 | [M+H]^+^ | 383.19/337.00 | 21 | 94 |

**Table S5.** The accession numbers of plant UGTs that are used for the phylogenetic

| **Group** | **Accession number** | **name** | **Species** |
| --- | --- | --- | --- |
| 1 | AAK28303.1 | Nt7GlcT | Nicotiana tabacum |
| 1 | BAA83484.1 | SbUF7GT | Scutellaria baicalensis |
| 1 | AAS94329.1 | UGT73A4 | Beta vulgaris |
| 1 | Q94C57 | UGT73B2 | Arabidopsis thaliana |
| 1 | AEC09298 | UGT73C6 | Arabidopsis thaliana |
| 1 | ABI94020.1 | UGT73C8 | Medicago truncatula |
| 1 | Q9LNE6 | UGT89C1 | Arabidopsis thaliana |
| 2 | AAL06646.2 | Cm1–2RhaT | Citrus maxima |
| 2 | BAH80312.1 | CrUGT3 | Catharanthus roseus |
| 2 | CAA50376 | Ph1-6RhaT(Petunia 3RT) | Petunia hybrida |
| 2 | I1LCI8 | UGT79A6 | Glycine max |
| 2 | BAD95882.1 | UGT79G16 | Ipomoea purpura |
| 2 | BAD77944.1 | UGT94B1 (BpUGAT) | Bellis perennis |
| 3 | AAD55985 | PhF3GlcT | Petunia hybrida |
| 3 | BAD06514.1 | UGT78A2 | Aralia cordata |
| 3 | NP_197205 | UGT78D1 | Arabidopsis thaliana |
| 3 | NP_197207 | UGT78D2 | Arabidopsis thaliana |
| 3 | A6XNC6 | UGT78G1 | Medicago truncatula |
| 3 | AAB81683 | VvGT1 | Vitis vinifera |
| 3 | BAI22846 | VvGT5 | Vitis vinifera |
| 3 | BAI22847 | VvGT6 | Vitis vinifera |
| 3 | CAA31856.1 | Zm3GlcT | Zea mays |
| 4 | A0A0B6VIJ5 | GtUF6CGT1 | Gentiana triflora |
| 4 | Q0WW21 | Pf5GlcT | Perilla frutescens |
| 4 | BAA89009.1 | PhA5GT | Petunia hybrida |
| 4 | BAC54093.1 | Th5GT | Torenia hybrida |
| 4 | BAA36423 | Vh5GlcT | Verbena hybrida |
| 5 | BAO79433 | GmUGT1 | Glycine max |
| 5 | BAO79434.1 | GmUGT3 | Glycine max |
| 5 | BAO79435.1 | GmUGT4 | Glycine max |
| 5 | BAO79436.1 | GmUGT7 | Glycine max |
| 5 | BAO79437.1 | GmUGT8 | Glycine max |
| 5 | BAO79438.1 | GmUGT9 | Glycine max |
| 6 | A0A0A1HA03 | FeUGT708C1 | Fagopyrum esculentum |
| 6 | A0A0A1H7N4 | FeUGT708C2 | Fagopyrum esculentum |
| 6 | ABI94020 | OsCGT | Oryza sativa |
| 6 | AMQ26115.2 | PlUGT43 | Pueraria lobata |
| 6 | I1L3T1 | GmCGT (UGT708D1) | Glycine max |
| 6 | LC131333 | UGT708G1 | Fortunella crassifolia |
| 6 | LC131334 | UGT708G2 | Citrus unshiu |
| 6 | NP_001132650 | ZmCGT (UGT708A6) | Zea mays |
| 6 | MK644229 | TcCGT | Trollius chinensis |
| 7 | AAN79728 | Iron | Escherichia coli |
| 7 | AAF00209 | UrdGT2 | Streptomyces fradiae |

**Table S6.** Molecular diversity and nucleotide diversity

|  |  | Tajima's D test | | | | | Fu's FS test | | | | | | - |
| --- | --- | --- | --- | --- | --- | --- | --- | --- | --- | --- | --- | --- | --- |
| Population | Sample size | S | Pi | Tajima's D | | Tajima's D  *P*-value | | No. of alleles | Theta  pi | Exp. no. of alleles | FS | FS  *P*-value | Nucleotide diversity (π, average over loci) |
| AHHS | 11 | 16 | 6.18182 | 0.58678 | | 0.753 | | 8 | 6.18182 | 6.6511 | -0.90707 | 0.262 | 0.071882 ± 0.041755 |
| FJGZ | 5 | 9 | 3.8 | -0.8554 | | 0.269 | | 4 | 3.8 | 3.49284 | 0.05125 | 0.373 | 0.044186 ± 0.031189 |
| FJTN | 6 | 6 | 3.2 | 1.24649 | | 0.903 | | 2 | 3.2 | 3.72811 | 4.18389 | 0.968 | 0.037209 ± 0.025757 |
| GDHY | 9 | 20 | 7 | -0.23974 | | 0.426 | | 7 | 7 | 6.0776 | -0.47234 | 0.33 | 0.081395 ± 0.047917 |
| GDLFS | 7 | 4 | 2 | 1.0756 | | 0.862 | | 3 | 2 | 3.43571 | 1.32063 | 0.762 | 0.023256 ± 0.017000 |
| GDSGRH | 12 | 12 | 5.31818 | 1.4214 | | 0.944 | | 6 | 5.31818 | 6.63938 | 1.18428 | 0.716 | 0.061839 ± 0.036151 |
| GDSX | 11 | 28 | 10.36364 | | 0.38845 | 0.658 | | 6 | 10.36364 | 7.76051 | 2.77011 | 0.886 | 0.120507 ± 0.067239 |
| GXBS | 5 | 1 | 0.6 | 1.22474 | | 0.946 | | 2 | 0.6 | 1.90287 | 0.62615 | 0.485 | 0.006977 ± 0.007643 |
| GXGC | 7 | 12 | 4.47619 | -0.46887 | | 0.357 | | 7 | 4.47619 | 4.53502 | -3.28606 | 0.02 | 0.052049 ± 0.033387 |
| GXGP | 7 | 14 | 5.52381 | -0.18346 | | 0.437 | | 7 | 5.52381 | 4.81316 | -2.77325 | 0.039 | 0.064230 ± 0.040247 |
| GXLP | 10 | 14 | 4.48889 | -0.42563 | | 0.348 | | 7 | 4.48889 | 5.62176 | -0.99856 | 0.261 | 0.052196 ± 0.031740 |
| GXRX | 10 | 14 | 4.37778 | -0.52846 | | 0.31 | | 9 | 4.37778 | 5.57074 | -4.0492 | 0.008 | 0.050904 ± 0.031051 |
| GXXA | 10 | 5 | 2.48889 | 1.6368 | | 0.962 | | 4 | 2.48889 | 4.44659 | 1.09267 | 0.736 | 0.028941 ± 0.019256 |
| GXYL | 4 | 21 | 11 | -0.40729 | | 0.489 | | 4 | 11 | 3.54853 | 0.44493 | 0.37 | 0.127907 ± 0.088286 |
| GZDS | 9 | 15 | 3.94444 | -1.37669 | | 0.094 | | 5 | 3.94444 | 5.05409 | 0.65645 | 0.622 | 0.045866 ± 0.028741 |
| GZSD | 9 | 8 | 2.05556 | -1.36919 | | 0.093 | | 3 | 2.05556 | 3.90354 | 1.85521 | 0.853 | 0.023902 ± 0.016727 |
| HBXN | 11 | 17 | 5.30909 | -0.38194 | | 0.358 | | 10 | 5.30909 | 6.30962 | -4.28831 | 0.011 | 0.061734 ± 0.036421 |
| HNPJ | 7 | 12 | 4.95238 | 0.0605 | | 0.553 | | 5 | 4.95238 | 4.67006 | 0.27587 | 0.515 | 0.057586 ± 0.036507 |
| HNXN | 7 | 6 | 2 | -0.93141 | | 0.251 | | 4 | 2 | 3.43571 | -0.13194 | 0.385 | 0.023256 ± 0.017000 |
| HNYY | 12 | 12 | 5.22727 | 1.32529 | | 0.934 | | 6 | 5.22727 | 6.59677 | 1.13714 | 0.712 | 0.060782 ± 0.035600 |
| HNRL | 2 | 10 | 10 | 0 | | 1 | | 2 | 10 | 1.90909 | 2.30259 | 0.53 | 0.116279 ± 0.121955 |
| JXGF | 10 | 15 | 5.37778 | 0.06558 | | 0.548 | | 9 | 5.37778 | 5.98885 | -3.34948 | 0.021 | 0.062532 ± 0.037246 |
| JXHC | 5 | 12 | 4.8 | -1.20539 | | 0.043 | | 4 | 4.8 | 3.69431 | 0.42469 | 0.491 | 0.055814 ± 0.038298 |
| JXLHS | 9 | 12 | 4.33333 | -0.0879 | | 0.473 | | 6 | 4.33333 | 5.22496 | -0.32044 | 0.409 | 0.050388 ± 0.031191 |
| JXYS | 8 | 13 | 5.60714 | 0.59928 | | 0.758 | | 8 | 5.60714 | 5.27729 | -3.58408 | 0.019 | 0.065199 ± 0.039876 |
| JXXG | 9 | 12 | 3.66667 | -0.8033 | | 0.236 | | 5 | 3.66667 | 4.92146 | 0.49185 | 0.586 | 0.042636 ± 0.026988 |
| JXXS | 6 | 11 | 4.4 | -0.52415 | | 0.341 | | 6 | 4.4 | 4.0888 | -2.4199 | 0.029 | 0.051163 ± 0.033914 |
| SCSM | 4 | 8 | 4.33333 | -0.06867 | | 0.607 | | 4 | 4.33333 | 3.08762 | -0.71498 | 0.16 | 0.050388 ± 0.037509 |
| YNGN | 13 | 21 | 7.30769 | 0.34286 | | 0.645 | | 12 | 7.30769 | 7.79897 | -4.77183 | 0.01 | 0.084973 ± 0.047823 |
| YNQJ | 5 | 14 | 8.2 | 1.60517 | | 0.963 | | 5 | 8.2 | 4.0995 | -0.6076 | 0.212 | 0.095349 ± 0.062353 |
| ZJLS | 6 | 10 | 4.13333 | -0.33767 | | 0.412 | | 4 | 4.13333 | 4.0193 | 0.82132 | 0.65 | 0.048062 ± 0.032106 |
| ZJQZ | 9 | 15 | 5 | -0.45381 | | 0.331 | | 7 | 5 | 5.484 | -1.23029 | 0.204 | 0.058140 ± 0.035383 |
| ZJYDS | 11 | 20 | 5.74545 | -0.71849 | | 0.244 | | 8 | 5.74545 | 6.48722 | -1.09443 | 0.241 | 0.066808 ± 0.039089 |
| ZJYK | 8 | 12 | 3.89 | -0.79932 | | 0.245 | | 7 | 3.89286 | 4.70284 | -2.52918 | 0.037 | 0.045266 ± 0.028903 |
| Mean | 8.05882 | 12.68 | 5.03 | -0.01729 | | 0.52 | | 5.76 | 5.03252 | 4.85229 | -0.52617 | 0.37979 | N.A. |
| s.d. | 2.67357 | 5.43 | 2.31 | 0.86209 | | 0.29 | | 2.36 | 2.31191 | 1.45418 | 2.13525 | 0.29517 | N.A. |

**Table S7**. (a) Population pairwise Fsts (Distance method: Pairwise difference)

|  | AHHS | FJGZ | FJTN | GDHY | GDLFS | GDSGRH | GDSX | GXBS | GXGC | GXGP | GXLP | GXRX |
| --- | --- | --- | --- | --- | --- | --- | --- | --- | --- | --- | --- | --- |
| AHHS | 0 |  |  |  |  |  |  |  |  |  |  |  |
| FJGZ | 0.217 | 0 |  |  |  |  |  |  |  |  |  |  |
| FJTN | 0.3 | 0.313 | 0 |  |  |  |  |  |  |  |  |  |
| GDHY | 0.295 | 0.263 | 0.277 | 0 |  |  |  |  |  |  |  |  |
| GDLFS | 0.407 | 0.528 | 0.482 | 0.295 | 0 |  |  |  |  |  |  |  |
| GDSGRH | 0.282 | 0.331 | 0.335 | 0 | 0.238 | 0 |  |  |  |  |  |  |
| GDSX | 0.239 | 0.271 | 0.311 | 0.262 | 0.424 | 0.359 | 0 |  |  |  |  |  |
| GXBS | 0.413 | 0.771 | 0.739 | 0.475 | 0.788 | 0.467 | 0.401 | 0 |  |  |  |  |
| GXGC | 0.242 | 0.06 | 0.262 | 0.142 | 0.36 | 0.178 | 0.279 | 0.651 | 0 |  |  |  |
| GXGP | 0.223 | 0.321 | 0.259 | 0.207 | 0.202 | 0.181 | 0.276 | 0.41 | 0.186 | 0 |  |  |
| GXLP | 0.317 | 0.249 | 0.29 | 0.225 | 0.304 | 0.221 | 0.356 | 0.575 | 0.143 | 0.164 | 0 |  |
| GXRX | 0.272 | 0.183 | 0.092 | 0.159 | 0.231 | 0.173 | 0.326 | 0.578 | 0.076 | 0.111 | 0.099 | 0 |
| GXXA | 0.434 | 0.474 | 0.324 | 0.228 | 0.411 | 0.234 | 0.423 | 0.705 | 0.302 | 0.241 | 0.248 | 0.087 |
| GXYL | 0.075 | 0.297 | 0.401 | 0.267 | 0.491 | 0.288 | 0.212 | 0.348 | 0.25 | 0.225 | 0.36 | 0.367 |
| GZDS | 0.286 | 0.35 | 0.208 | 0.196 | 0.175 | 0.159 | 0.344 | 0.504 | 0.187 | 0.018 | 0.129 | 0 |
| GZSD | 0.365 | 0.477 | 0.36 | 0.258 | 0.257 | 0.208 | 0.389 | 0.672 | 0.244 | 0.059 | 0.162 | 0.052 |
| HBXN | 0.226 | 0.164 | 0.12 | 0.125 | 0.222 | 0.135 | 0.287 | 0.496 | 0.004 | 0.089 | 0.107 | 0 |
| HNPJ | 0.017 | 0.366 | 0.475 | 0.305 | 0.569 | 0.311 | 0.248 | 0.488 | 0.302 | 0.321 | 0.413 | 0.409 |
| HNXN | 0.436 | 0.563 | 0.426 | 0.295 | 0.417 | 0.276 | 0.405 | 0.699 | 0.365 | 0.143 | 0.249 | 0.127 |
| HNYY | 0.224 | 0.345 | 0.298 | 0.246 | 0.308 | 0.224 | 0.285 | 0.328 | 0.234 | 0.051 | 0.229 | 0.202 |
| HNRL | 0.176 | 0.059 | 0.082 | 0.088 | 0.379 | 0.177 | 0.141 | 0.7 | 0 | 0.086 | 0.132 | 0.01 |
| JXGF | 0.171 | 0.11 | 0.086 | 0.21 | 0.247 | 0.225 | 0.285 | 0.515 | 0.106 | 0.109 | 0.135 | 0 |
| JXHC | 0.253 | 0.32 | 0.239 | 0.134 | 0.238 | 0.118 | 0.275 | 0.481 | 0.12 | 0 | 0.09 | 0.007 |
| JXLHS | 0.382 | 0.327 | 0.2 | 0.368 | 0.489 | 0.411 | 0.381 | 0.671 | 0.367 | 0.339 | 0.365 | 0.227 |
| JXQS | 0.227 | 0 | 0.127 | 0.249 | 0.417 | 0.312 | 0.281 | 0.631 | 0.11 | 0.266 | 0.231 | 0.109 |
| JXXG | 0.251 | 0.089 | 0.292 | 0.224 | 0.502 | 0.287 | 0.29 | 0.698 | 0.001 | 0.298 | 0.258 | 0.189 |
| JXXS | 0.111 | 0.458 | 0.573 | 0.417 | 0.687 | 0.449 | 0.274 | 0.64 | 0.431 | 0.454 | 0.528 | 0.528 |
| SCSM | 0.302 | 0.414 | 0.33 | 0.177 | 0.388 | 0.175 | 0.288 | 0.536 | 0.227 | 0.065 | 0.172 | 0.11 |
| YNGN | 0.042 | 0.199 | 0.241 | 0.164 | 0.292 | 0.141 | 0.218 | 0.177 | 0.136 | 0.075 | 0.189 | 0.168 |
| YNQJ | 0.155 | 0.272 | 0.294 | 0.181 | 0.327 | 0.161 | 0.252 | 0.321 | 0.155 | 0.093 | 0.201 | 0.185 |
| ZJLS | 0.108 | 0.039 | 0.132 | 0.21 | 0.465 | 0.282 | 0.21 | 0.687 | 0.076 | 0.221 | 0.235 | 0.092 |
| ZJQZ | 0.148 | 0.109 | 0.016 | 0.215 | 0.358 | 0.257 | 0.257 | 0.572 | 0.13 | 0.176 | 0.204 | 0.026 |
| ZJYDS | 0.124 | 0 | 0.105 | 0.21 | 0.289 | 0.239 | 0.265 | 0.544 | 0.042 | 0.166 | 0.153 | 0.04 |
| ZJYK | 0.204 | 0.078 | 0.314 | 0.144 | 0.374 | 0.16 | 0.285 | 0.656 | 0 | 0.203 | 0.163 | 0.136 |

**Table S7**. (b) Population pairwise Fsts (Distance method: Pairwise difference)

|  | GXXA | GXYL | GZDS | GZSD | HBXN | HNPJ | HNXN | HNYY | HNRL | JXGF | JXHC | JXLHS |
| --- | --- | --- | --- | --- | --- | --- | --- | --- | --- | --- | --- | --- |
| GXXA | 0 |  |  |  |  |  |  |  |  |  |  |  |
| GXYL | 0.504 | 0 |  |  |  |  |  |  |  |  |  |  |
| GZDS | 0.099 | 0.347 | 0 |  |  |  |  |  |  |  |  |  |
| GZSD | 0.18 | 0.444 | 0 | 0 |  |  |  |  |  |  |  |  |
| HBXN | 0.148 | 0.272 | 0.02 | 0.059 | 0 |  |  |  |  |  |  |  |
| HNPJ | 0.56 | 0 | 0.414 | 0.511 | 0.31 | 0 |  |  |  |  |  |  |
| HNXN | 0.231 | 0.476 | 0.047 | 0.06 | 0.16 | 0.571 | 0 |  |  |  |  |  |
| HNYY | 0.307 | 0.242 | 0.139 | 0.168 | 0.151 | 0.281 | 0.224 | 0 |  |  |  |  |
| HNRL | 0.288 | 0.092 | 0.108 | 0.278 | 0 | 0.298 | 0.361 | 0.157 | 0 |  |  |  |
| JXGF | 0.184 | 0.301 | 0.057 | 0.127 | 0.017 | 0.34 | 0.213 | 0.187 | 0.005 | 0 |  |  |
| JXHC | 0.121 | 0.234 | 0 | 0 | 0 | 0.345 | 0 | 0.074 | 0.023 | 0.065 | 0 |  |
| JXLHS | 0.384 | 0.472 | 0.302 | 0.405 | 0.254 | 0.53 | 0.428 | 0.36 | 0.152 | 0.167 | 0.314 | 0 |
| JXQS | 0.329 | 0.323 | 0.251 | 0.342 | 0.123 | 0.363 | 0.405 | 0.3 | 0 | 0.05 | 0.23 | 0.096 |
| JXXG | 0.404 | 0.292 | 0.313 | 0.396 | 0.11 | 0.302 | 0.486 | 0.301 | 0.088 | 0.192 | 0.242 | 0.39 |
| JXXS | 0.672 | 0 | 0.551 | 0.652 | 0.434 | 0 | 0.691 | 0.402 | 0.411 | 0.452 | 0.494 | 0.598 |
| SCSM | 0.26 | 0.252 | 0.027 | 0.087 | 0.06 | 0.401 | 0.126 | 0.132 | 0.111 | 0.144 | 0 | 0.366 |
| YNGN | 0.274 | 0 | 0.127 | 0.179 | 0.116 | 0.035 | 0.126 | 0.079 | 0.071 | 0.134 | 0.064 | 0.337 |
| YNQJ | 0.307 | 0 | 0.127 | 0.194 | 0.124 | 0.175 | 0.126 | 0.133 | 0.026 | 0.17 | 0.033 | 0.371 |
| ZJLS | 0.371 | 0.245 | 0.245 | 0.379 | 0.067 | 0.274 | 0.126 | 0.247 | 0.014 | 0.007 | 0.233 | 0.27 |
| ZJQZ | 0.24 | 0.292 | 0.135 | 0.24 | 0.047 | 0.314 | 0.126 | 0.216 | 0 | 0 | 0.143 | 0.085 |
| ZJYDS | 0.261 | 0.267 | 0.152 | 0.219 | 0.055 | 0.273 | 0.126 | 0.218 | 0 | 0 | 0.14 | 0.202 |
| ZJYK | 0.337 | 0.235 | 0.214 | 0.286 | 0.063 | 0.255 | 0.126 | 0.233 | 0.068 | 0.144 | 0.162 | 0.415 |

**Table S7**. Population pairwise Fsts (Distance method: Pairwise difference)

|  | JXQS | JXXG | JXXS | SCSM | YNGN | YNQJ | ZJLS | ZJQZ | ZJYDS | ZJYK |
| --- | --- | --- | --- | --- | --- | --- | --- | --- | --- | --- |
| JXQS | 0 |  |  |  |  |  |  |  |  |  |
| JXXG | 0.114 | 0 |  |  |  |  |  |  |  |  |
| JXXS | 0.435 | 0.405 | 0 |  |  |  |  |  |  |  |
| SCSM | 0.296 | 0.36 | 0.54 | 0 |  |  |  |  |  |  |
| YNGN | 0.214 | 0.176 | 0.155 | 0.096 | 0 |  |  |  |  |  |
| YNQJ | 0.256 | 0.248 | 0.28 | 0.074 | 0.004 | 0 |  |  |  |  |
| ZJLS | 0 | 0.038 | 0.376 | 0.325 | 0.12 | 0.213 | 0 |  |  |  |
| ZJQZ | 0 | 0.127 | 0.413 | 0.217 | 0.142 | 0.197 | 0 | 0 |  |  |
| ZJYDS | 0 | 0.074 | 0.37 | 0.219 | 0.13 | 0.187 | 0 | 0 | 0 |  |
| ZJYK | 0.151 | 0 | 0.393 | 0.277 | 0.1 | 0.162 | 0.084 | 0.157 | 0.048 | 0 |

**Table S8**. Haplotype kinds and numbers of 34 populations

| Area | Pn | Hap | Hn | Area | Pn | Hap | Hn | Area | Pn | Hap | Hn | Area | Pn | Hap | Hn |
| --- | --- | --- | --- | --- | --- | --- | --- | --- | --- | --- | --- | --- | --- | --- | --- |
| AHHS | 11 | Hap21 | 1 | GXLP | 10 | Hap8 | 4 | HNYY | 12 | Hap3 | 3 | YNGN | 13 | Hap52 | 1 |
|  |  | Hap22 | 3 |  |  | Hap16 | 1 |  |  | Hap7 | 5 |  |  | Hap53 | 1 |
|  |  | Hap55 | 1 |  |  | Hap38 | 1 |  |  | Hap35 | 1 |  |  | Hap91 | 1 |
|  |  | Hap69 | 1 |  |  | Hap97 | 1 |  |  | Hap129 | 1 |  |  | Hap92 | 1 |
|  |  | Hap70 | 2 |  |  | Hap102 | 1 |  |  | Hap130 | 1 |  |  | Hap93 | 1 |
|  |  | Hap71 | 1 |  |  | Hap103 | 1 |  |  | Hap131 | 1 |  |  | Hap39 | 2 |
|  |  | Hap72 | 1 |  |  | Hap113 | 1 | JXGF | 10 | Hap1 | 2 |  |  | Hap49 | 1 |
|  |  | Hap73 | 1 | GXRX | 10 | Hap1 | 2 |  |  | Hap2 | 1 |  |  | Hap50 | 1 |
| FJGZ | 5 | Hap1 | 1 |  |  | Hap3 | 1 |  |  | Hap89 | 1 |  |  | Hap51 | 1 |
|  |  | Hap69 | 2 |  |  | Hap16 | 1 |  |  | Hap23 | 1 |  |  | Hap69 | 1 |
|  |  | Hap70 | 1 |  |  | Hap19 | 1 |  |  | Hap24 | 1 |  |  | Hap89 | 1 |
|  |  | Hap82 | 1 |  |  | Hap58 | 1 |  |  | Hap36 | 1 |  |  | Hap90 | 1 |
| FJTN | 6 | Hap27 | 4 |  |  | Hap59 | 1 |  |  | Hap56 | 1 | SCSM | 4 | Hap45 | 1 |
|  |  | Hap84 | 2 |  |  | Hap69 | 1 |  |  | Hap69 | 1 |  |  | Hap46 | 1 |
| GDHY | 9 | Hap1 | 1 |  |  | Hap70 | 1 |  |  | Hap70 | 1 |  |  | Hap47 | 1 |
|  |  | Hap11 | 1 |  |  | Hap85 | 1 | JXHC | 5 | Hap3 | 2 |  |  | Hap48 | 1 |
|  |  | Hap12 | 2 | GXXA | 10 | Hap3 | 2 |  |  | Hap4 | 1 | ZJLS | 6 | Hap1 | 2 |
|  |  | Hap125 | 1 |  |  | Hap28 | 3 |  |  | Hap5 | 1 |  |  | Hap70 | 1 |
|  |  | Hap126 | 2 |  |  | Hap60 | 1 |  |  | Hap95 | 1 |  |  | Hap77 | 2 |
|  |  | Hap127 | 1 |  |  | Hap61 | 4 | JXLHS | 9 | Hap23 | 1 |  |  | Hap81 | 1 |
|  |  | Hap128 | 1 | GXYL | 4 | Hap3 | 1 |  |  | Hap25 | 1 | ZJQZ | 9 | Hap23 | 1 |
| GDLFS | 7 | Hap10 | 3 |  |  | Hap91 | 1 |  |  | Hap26 | 4 |  |  | Hap1 | 1 |
|  |  | Hap37 | 2 |  |  | Hap111 | 1 |  |  | Hap57 | 1 |  |  | Hap22 | 1 |
|  |  | Hap9 | 2 |  |  | Hap112 | 1 |  |  | Hap67 | 1 |  |  | Hap26 | 2 |
| GDSGRH | 12 | Hap12 | 6 | GZDS | 9 | Hap3 | 4 |  |  | Hap68 | 1 |  |  | Hap70 | 1 |
|  |  | Hap65 | 1 |  |  | Hap17 | 2 | JXYS | 8 | Hap23 | 1 |  |  | Hap77 | 2 |
|  |  | Hap114 | 1 |  |  | Hap18 | 1 |  |  | Hap25 | 1 |  |  | Hap98 | 1 |
|  |  | Hap115 | 2 |  |  | Hap39 | 1 |  |  | Hap70 | 1 | ZJYDS | 11 | Hap1 | 2 |
|  |  | Hap116 | 1 |  |  | Hap121 | 1 |  |  | Hap82 | 1 |  |  | Hap23 | 1 |
|  |  | Hap117 | 1 | GZSD | 9 | Hap3 | 6 |  |  | Hap83 | 1 |  |  | Hap36 | 2 |
| GDSX | 11 | Hap4 | 1 |  |  | Hap44 | 2 |  |  | Hap1 | 1 |  |  | Hap69 | 1 |
|  |  | Hap31 | 1 |  |  | Hap95 | 1 |  |  | Hap69 | 1 |  |  | Hap74 | 2 |
|  |  | Hap122 | 6 | HBXN | 11 | Hap3 | 1 |  |  | Hap70 | 1 |  |  | Hap75 | 1 |
|  |  | Hap124 | 1 |  |  | Hap29 | 1 | JXXG | 9 | Hap26 | 1 |  |  | Hap76 | 1 |
|  |  | Hap66 | 1 |  |  | Hap30 | 1 |  |  | Hap78 | 4 |  |  | Hap77 | 1 |
|  |  | Hap123 | 1 |  |  | Hap31 | 1 |  |  | Hap99 | 1 | ZJYK | 8 | Hap1 | 1 |
| BXBS | 5 | Hap14 | 3 |  |  | Hap62 | 1 |  |  | Hap100 | 1 |  |  | Hap3 | 1 |
|  |  | Hap15 | 2 |  |  | Hap63 | 1 |  |  | Hap101 | 2 |  |  | Hap69 | 1 |
| GXGC | 7 | Hap1 | 1 |  |  | Hap64 | 1 | JXXS | 6 | Hap105 | 1 |  |  | Hap78 | 1 |
|  |  | Hap13 | 1 |  |  | Hap78 | 2 |  |  | Hap106 | 1 |  |  | Hap94 | 2 |
|  |  | Hap69 | 1 |  |  | Hap79 | 1 |  |  | Hap107 | 1 |  |  | Hap95 | 1 |
|  |  | Hap85 | 1 |  |  | Hap80 | 1 |  |  | Hap108 | 1 |  |  | Hap96 | 1 |
|  |  | Hap86 | 1 | HNPJ | 7 | Hap3 | 1 |  |  | Hap109 | 1 | GXGP | 7 | Hap3 | 1 |
|  |  | Hap87 | 1 |  |  | Hap34 | 1 |  |  | Hap110 | 1 |  |  | Hap40 | 1 |
|  |  | Hap88 | 1 |  |  | Hap116 | 3 | YNQJ | 5 | Hap3 | 1 |  |  | Hap41 | 1 |
| HNXN | 7 | Hap3 | 2 |  |  | Hap118 | 1 |  |  | Hap32 | 1 |  |  | Hap42 | 1 |
|  |  | Hap19 | 3 |  |  | Hap119 | 1 |  |  | Hap33 | 1 |  |  | Hap43 | 1 |
|  |  | Hap20 | 1 | HNRL | 2 | Hap6 | 1 |  |  | Hap91 | 1 |  |  | Hap44 | 1 |
|  |  | Hap54 | 1 |  |  | Hap104 | 1 |  |  | Hap120 | 1 |  |  | Hap132 | 1 |

Pn: the numbers of population; Hap: the kinds (names) of haplotype; Hn: the numbers of haplotype
